# *Operando* spectroelectrochemistry of bulk-exfoliated 2D SnS_2_ for anodes within alkali metal ion batteries reveals unusual tin (III) states

**DOI:** 10.3389/fchem.2022.1038327

**Published:** 2022-10-21

**Authors:** Mariusz Radtke, Christian Hess

**Affiliations:** Eduard Zintl Institute of Inorganic and Physical Chemistry, Technical University of Darmstadt, Darmstadt, Germany

**Keywords:** 2D anode, SnS_2_, *Operando* Raman, TMD, alkali-ion-batteries, impedance spectroscopy

## Abstract

In this study we report an affordable synthesis and preparation of an electrochemically exfoliated few-layer 2-dimensional (2D) SnS_2_ anode material of high cycling durability and demonstrate its performance on the example of alkali metal batteries. The metalation mechanism consists of highly unusual and previously only speculated Sn (III)-state grasped by *operando* Raman spectroelectrochemistry aided by symmetry analysis. The prepared 2D material flakes were characterized by high resolution transmission electron microscopy, X-ray photoelectron and Raman spectroscopies. The *operando* Raman spectroelectrochemistry was chosen as a dedicated tool for the investigation of alkali-metal-ion intercalation (Li, Na, K), whereby the distortion of the A_1g_ Raman active mode (out-of-plane S-Sn-S vibration) during battery charging exhibited a substantial dependence on the electrochemically applied potential. As a result of the structural dynamics a considerable Raman red-shift of 17.6 cm^−1^ was observed during metalation. Linewidth changes were used to evaluate the expansion caused by metalation, which in case of sodium and potassium were found to be minimal compared to lithium. Based on the spectroscopic and electrochemical results, a mechanism for the de-/intercalation of lithium, sodium and potassium is proposed which includes alloying in few-layer 2D SnS_2_ materials and the generation of point-defects.

## 1 Introduction

The importance of 2D materials in energy storage is constantly growing, allowing for unique conduction mechanisms (Pomerantseva and Gogotsi, 2017). The mass-transport driven conductivity in lower-dimensional materials possessing a band-gap in semiconductor range (in case of SnS_2_ 2.3 eV) utilizes dedicated channels between the sheets for ionic movements, rather than penetrating the whole bulk of a material in stochastic and random fashion, as it is the case for still more common 3D materials (Yang et al., 2013). The use of the channels releases the stress from the material, which results in little to no expansion during the metalation. By dedicated use of 2D materials with conductivity mechanisms allowing to avoid structural expansion, as well as by alloying them with alkali metals for higher cycling stability, the manufacturing of future-generation batteries becomes an achievable goal.

An example of such materials, which exhibit both relatively wide band-gap, 2D structures and fine Raman cross-section (with strong dependence on number of layers) are transition-metal-dichalcogenides (TMD) and their close-related analogues not-containing the transition metals e.g. SnS_2_ (Zhang et al., 2015). As 3-dimensional material SnS_2_ was already widely applied as an anode material, while due to its unique conduction mechanism in 2D it is currently a matter of intensive research. Most of the mechanistic research reports do not mention the occurrence of unusual oxidation states within tin, where Sn (IV) and Sn (II) are the most common ones. The types of interaction of alkali metal ions with tin, which can lead to highly unusual oxidation states like Sn (III), might have important implications in understanding energy storage within the anode on a molecular level. The presence of a Sn (III) state generated electrochemically has been proposed previously based on kinetic studies on “very thin materials”, prior to today’s era of intensive investigation of 2D materials (Chang and Bard, 2014).

In addition, as the energy production is less of a challenge nowadays due to renewable and sustainable sources, its storage in reliable reposition media connected to smart grids is a matter of highest importance during the so-called energy-change (Koohi-Fayegh and Rosen, 2020) (Kolokotsa et al., 2019). The understanding of reaction mechanisms within energy storage materials on an atomic level is crucial for future developments allowing to increase cyclic stability, reduction of waste and negative contributions to the carbon cycle (Kolokotsa et al., 2019). The need of replacing lithium with low-cost and ubiquitous sodium/potassium is a highly sought solution. The main drawback of using lower-dimensional materials in energy research is their relatively high cost of production (Velický and Toth, 2017).

For sodium and potassium-ion batteries the crystallographic expansion is the most common obstacle and reaches up to 400% when silicon is used as an anode (Mukhopadhyay and Sheldon, 2014). The expansion as a direct result of alloy formation of e.g. sodium with silicon, leading to considerable cracking, and therefore making the battery unusable over reasonable charge/discharge cycles. On the other hand, more pronounced reversibility of cycling was found to be reached by alloying sodium or potassium metals with oxides like cubic CeO_2_, rather than intercalating them (Yanjiao et al., 2020).

Vibrational Raman spectroscopy is a versatile technique with great potential for mechanistic investigations, allowing to investigate cumulative changes caused by the peculiarities of battery operation in an *operando* manner, such as chemical reactions, intercalation, alloying, reactions with the electrolyte, and build-up of passivating layers due to the electrolyte decomposition. Other techniques like *operando* XRD (X-Ray Diffraction) focus rather on crystallographic structure and changes induced by varying the potential, while *operando* Raman spectroelectrochemistry allows to observe the (local) vibrational structure of the electrode/electrolyte system. On the other hand, *operando* X-Ray Absorption (XAS) provides valuable structural information, but usually requires sophisticated synchrotron light sources. Therefore, *operando* Raman spectroelectrochemistry is a technique allowing in-depth structural insight, which is easily accessible in a non-specialized laboratory environment. As for now, transmission electron microscopy (TEM) is able to investigate batteries only *in-situ* (Woods et al., 2019).

SnS_2_ in its 2H and 1T polytypes belongs to a modern class of 2-dimensional layered chalcogenides (2D LC) with relatively wide indirect bandgap (∼2.3 eV) and is therefore a highly sought anode material for modern lithium-ion batteries (Ali et al., 2020) (Wang et al., 2016). The layered 2D structure of SnS_2_ is promising for exfoliation (either mechanical, sono- or electrochemical) and the indirect band-gap in the semiconductor range makes SnS_2_ a potentially more lucrative material as compared to graphene for achieving large specific capacities (Chaves et al., 2020) (Duan et al., 2015).

A plethora of examples utilizing 2D materials as anodes and cathodes was already reported, nevertheless a detailed understanding of the changes occurring during the charging of a lithium-ion-battery (LIB) and recently emerging sodium-ion-batteries (SIB)/potassium-ion-batteries (KIB) is still missing (Rojaee and Shahbazian-Yassar, 2020) (Shi and Zhao, 2017) (Gullapalli et al., 2017) (Zuo et al., 2020) (Peng et al., 2020). The main drawback for industrial applications is their high price, reaching several thousand Euros/Dollars per crystal. On the other hand, electrochemical exfoliation from the bulk by anodic and cathodic processes was reported by others (Magda et al., 2015) (Eng et al., 2014).

In our study, we present an affordable solution for the high-price obstacle towards commercialization by using clean electrochemical mixed anodic and cathodic exfoliation from bulk pressed pellets of SnS_2_ as block electrode and platinum wire as ground electrode. Thus, prepared few-layered 2D SnS_2_ materials were investigated towards their lithiation, sodiation and potassiation mechanisms employing a specially designed spectroelectrochemical battery cell equipped with borosilicate window allowing laser penetration into the anode interior. Supported by additional electrochemical and spectroscopic analysis an intercalation mechanism for alkali metals in 2D layered SnS_2_ materials is proposed, including an intermediate Sn(III)-oxidation state evidenced during *operando* cycling, as well as defect formation as part of the alloying reactions between SnS_2_ and lithium, sodium or potassium.

## 2 Materials and methods

### 2.1 Synthesis

**FIGURE 1:**
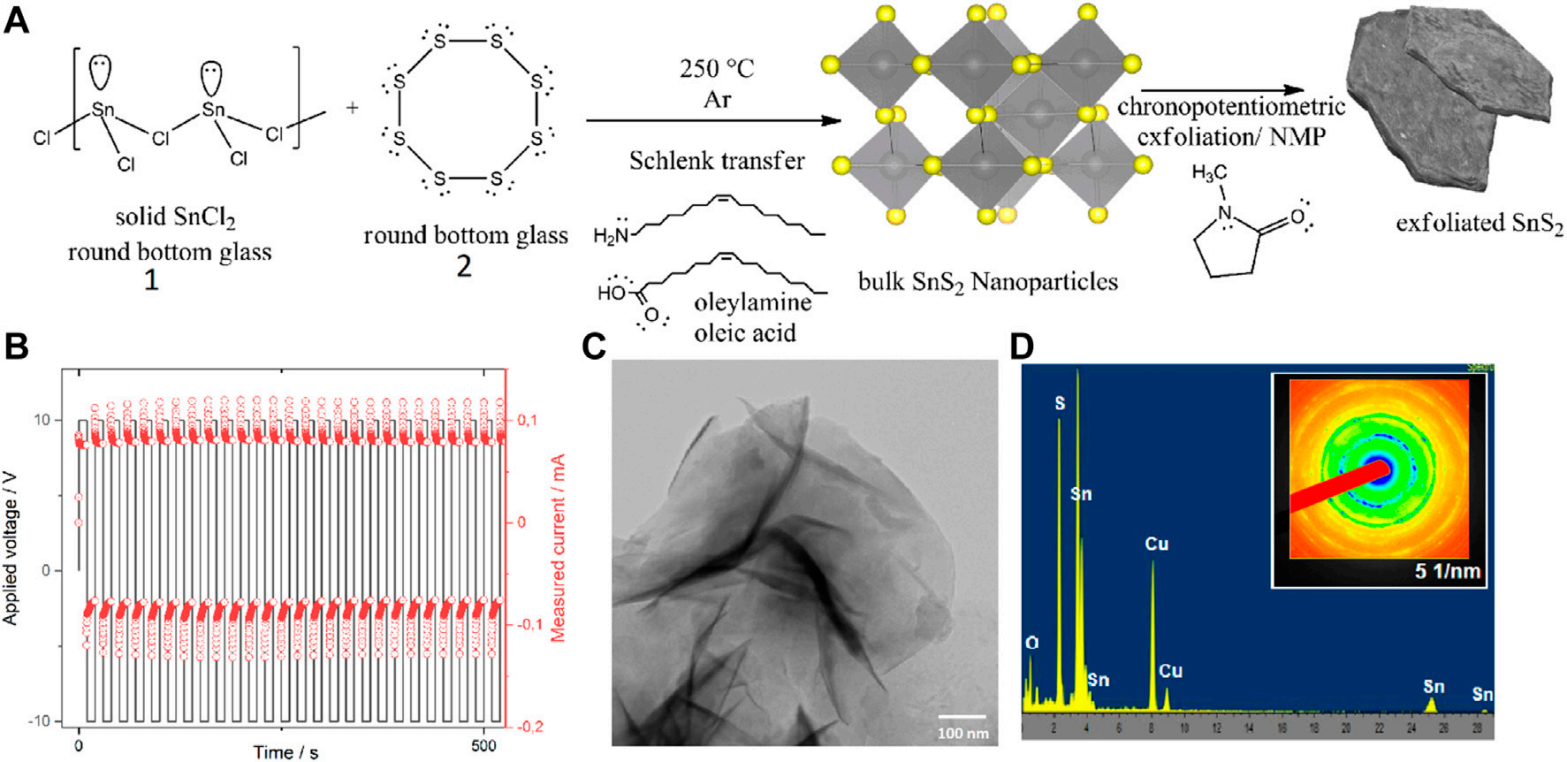
Synthesis and characterization of 2D layered SnS_2_. **(A)** Reaction scheme and **(B)** potential/current/time relation during the mixed character exfoliation (each cycle consists of single square wave). **(C)** TEM bright-field image with dark diffraction spots and **(D)** SAED pattern of prepared SnS_2_ with an inset image of the exfoliated SnS_2_ in water (which prior the exfoliation was unable to get dispersed). For details see text.

**FIGURE 2:**
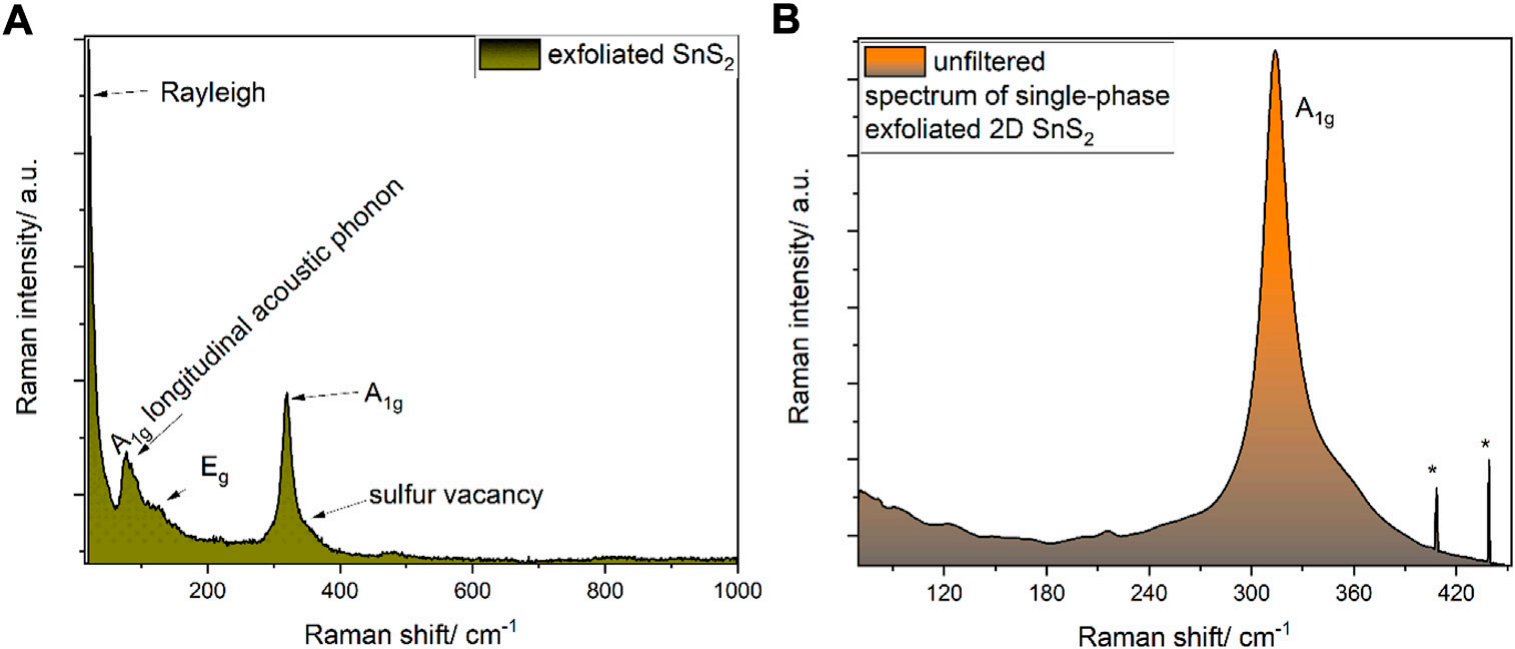
**(A)** Raman spectrum (633 nm) of the exfoliated SnS_2_. Please note the low position of the longitudinal acoustic phonons, which are located much lower than the modes observed during the exfoliation in e.g. Supplementary Figure S7. **(B)** Raman spectrum (633 nm) of 2D SnS_2_. Asterisks (*) represent cosmic rays signals. The shoulder at around 330 cm^−1^ is attributed to a S^2−^ vacancy. For detailed analysis see Supplementary Figure S8, as discussed in the text.

Single phase 2H-SnS_2_ was synthesized according to the procedure described elsewhere, which was slightly modified (Thangaraju et al., 2020). Briefly, 10 mmol of tin dichloride (99.8% purity, Merck/Sigma Aldrich) was placed in a three-neck round-bottom flask charged with a magnetic stirrer (Figure 1, inset A, “round bottom glass 1”), while a second round-bottom flask (Figure 1, inset A, “round-bottom glass 2”) was charged with sulfur powder (99.9% purity, Alfa Aesar, United Kingdom). To each flask, 1 ml of oleylamine (75%, Merck/Sigma Aldrich, Germany) and 1 ml of oleic acid (99.9% purity, ampule, Merck/Sigma Aldrich) were added. Each suspension was evacuated and re-filled with dry-nitrogen three times in a Schlenk-line. Furthermore, the flasks were heated up to 140 °C under vacuum in order to remove any built-up moisture and subsequentially heated up to 250 °C in a in house-made sand-bath for heat-transfer stability. After a temperature of 250°C was reached, the suspensions turned into clear solutions and at this temperature, by using a Schlenk-cannula transfer technique, the sulfur suspension was added drop-wise to the tin dichloride solution in oleylamine/oleic acid. The evolution of gas was observed and attributed to hydrochloric acid (according to the mechanism outlined in Supplementary Figure S1). The solution was kept for 60 min at this temperature, cooled down to room temperature and washed four times with absolute ethanol by centrifugation (7871 rcf, 10 min each time) and finally stored in dry n-hexane (Merck/Sigma Aldrich, Germany, self-dried over 4Å molecular sieves). The presence of single phase 2H SnS_2_ was confirmed by means of Raman spectroscopy (Figures 2 and Supplementary Figure S2) revealing the presence of a single A_1g_ mode at 316 cm^−1^ with no side band corresponding to the other polytypes (Thangaraju et al., 2020). The 2H-SnS_2_ material was pressed onto an Al_2_O_3_ grid (0.48 mm thick aluminium, mesh 0.016, Paco GmbH, Germany) under 9440 psi of pressure (10t Specac FTIR/XRF hydraulic press, United Kingdom). The pellet was immersed in 10 ml of N-methyl-2-pyrrolidone (NMP, 99% purity, Merck/Sigma Aldrich, Germany) and a series of chronopotentiometric pulses of ± 10 V every 10 s with current acquisition was applied for 2 h (Figure 1B). After already 10 cycles the previously coagulated NMP 2H-SnS_2_ material started to transfer into the organic phase. The solution was centrifugated at 7871 rcf, decanted and re-dispersed in dry n-hexane. The so-generated nanomaterial was subjected to further micro- and spectroscopic characterization by transmission electron microscopy (TEM) (Figures 1C,D), Raman and X-ray photoelectron (XP) spectroscopies as well as powder X-ray diffraction (XRD) (Supplementary Figure S3).

### 2.2 X-ray photoelectron spectroscopy

X-ray photoelectron spectra were acquired on a SSX100 ESCA Spectrometer (Surface Science), using monochromatic X-rays from the Al K-α line (9 kV, 10 mA) and a 0.25 × 1.0 mm measurement spot. The resolution was set as 0.16 eV and the acquisition rate as 0.5 eV. The base pressure of the analysis chamber was 2.8E-08 Torr. The spectrometer was calibrated with a gold foil (99.99% purity, Alfa Aesar, United Kingdom) using the Au4f_7/2_ photoemission. All XP spectra were quantified by Casa XPS (version 2.3.22PR1.0), referring to the residual carbon C1s signal (284.5 eV).

### 2.3 Raman spectroscopy

Raman spectra were acquired on a Jobin-Yvon Horiba Lab-Ram HRS 800 (10% radiation filter, 600 lines/mm grid, slit 150, hole: 1000), using 633 nm excitation from a He-Ne laser and a ×100 objective (Olympus, NA = 0.95). The excitation power was set to 1.2 mW. The laser spot was microscopically determined to be 1.3 µm in diameter and the acquisition time for each spectrum was set as 60 s. The *operando* spectroelectrochemistry was conducted in the same fashion, but by using a long-working distance objective (Olympus ×50 LWD, NA 0.55, MPLN). Prior each measurement, the stability of the experimental conditions was checked by acquisition of time-resolved spectra over 30 min with the same laser acquisition power and optics in order to exclude laser-induced effects like photobleaching (Supplementary Figure S4). These studies were performed at open circuit potential (OCP), while laser constantly acquiring Raman spectra, without variation in potential. The full-width at half maximum (FWHM) analysis was performed on normalized Raman spectra. Please note that each spectrum was fitted individually by Voigt functions.

### 2.4 (Spectro-) Electrochemical analysis

Electrochemical measurements including differential pulse voltammetry (DPV), alternating current cyclic voltammetry and staircase potentio-electrochemical impedance spectroscopy (SPEIS) were recorded on a BioLogic VSP potentiostat equipped with a PC-controlled EC-Lab software (Version 11.33). The differential pulse voltammetry spectra were acquired in a 2-electrode ECC-Opto-Std cell configuration in an optical spectroelectrochemical cell from EL-Cell GmbH, Hamburg, Germany.

The scanning window was set as 1.8–4.5 V vs. Li/Li^+^, and adapted to 1.49–4.19 V vs. Na/Na^+^, and 1.71–4.41 V vs. K/K^+^, in order to keep the potential windows comparable during spectroelectrochemical scans (Li/Li^+^ = −3.022 V vs. SHE, Na/Na^+^ = −2.710 V vs. SHE, K/K^+^ = −2.930 V vs. SHE). The pulse height was set as 100 mV, the pulse width as 0.2 ms, the step height as 0.1 mV, the step time as 333 ms, and the scan rate as 0.3 mV/s. This translates to Raman spectra acquired every 9 mV during the *operando* spectroelectrochemical scan. The Tafel plot was acquired on a relaxed cell in the same potential range, while potentiostatic intermittent titration technique (PITT) was performed over the time course of 72 h. All assembly operations and electrolyte preparations (LiPF_6_, NaPF_6_, KPF_6_ and propylene carbonate (PC), purchased in highest purity from Merck/Sigma Aldrich, Germany, were performed under inert atmosphere of argon (M-Braun glove-box). Sodium and potassium in mineral oil were purchased from Merck/Sigma Aldrich, Germany, and were washed with propylene carbonate (anhydrous) under argon atmosphere prior punching out the electrodes. For *operando* Raman spectroelectrochemical experiments the positive electrode was composed of a metallic Li/Na/K strip, and the negative electrode was the 2D SnS_2_ pressed onto Al_2_O_3_ mesh. TimrexTM graphite, optimized for battery research, manufactured by Imerys, Switzerland, was used as conductive carbon; no binder was used (see below). In case of CV, DPV and PITT, the cathodic scan was performed first, followed by an anodic experiment. A Whatman filter paper served as separator, while 1M MPF_6_ (M = Li, Na, K) in propylene carbonate was used as electrolyte.

SPEI spectra were acquired with a single sine signal mode excitation within 1.8–4.5 V vs. Li/Li^+^, 1.49–4.19V vs. Na/Na^+^, and 1.71V - 4.41 vs. K/K^+^ scanning windows with 1399 potential steps, deliberately chosen in sufficiently large number to grasp transient changes. The current was acquired every 0.1 s within a frequency range from 1MHz to 1 mHz with 10 points per decade in logarithmic spacing. The amplitude of the sine wave was set as 10 mV with 475 waiting periods between each frequency measurement (with no averaging of the points). The scanning interval was set as dE = 300 μV, while each scan took 1 h and 48 min to complete. Fitting of SPEIS spectra was performed with the impedance equivalent-circuit Z-fit methodology provided by the EC-Lab (v.11.33) interface till the model successfully converged with a Levenberg-Marquardt fitting, 50.000 numerical iterations, and the weighting set as 1.

### 2.5 Transmission electron microscopy

The bright-field transmission electron microscopy (BFTEM) images and selected area electron diffraction (SAED) were acquired on a JEOL JEM 2100F equipped with a FEG, operating at 200 kV. For TEM grid preparation, a small amount of sample was dispersed in ethanol; 3–4 droplets of the suspension were applied on a carbon coated Cu-grid (Plano GmbH, Germany) by the sessile drop-method and allowed to dry. Energy-dispersive X-ray spectroscopy (EDX) was acquired with an EDAX Octane Plus SDD EDX within the JEOL JEM 2100F system.

### 2.6 X-ray diffraction

The X-ray diffractometer used for characterization of SnS_2_ flakes was manufactured by STOE (model Stadi P) and equipped with a Cu Kα1 (1.54056 Å) X-ray source. The Rietveld-analysis was performed with help of the freeware Profex 5.0.1 (release version 13 March 2022) (Döbelin and Kleeberg, 2015).

## 3 Results and discussion

### 3.1 Synthesis and characterization of SnS_2_

The wet-chemical synthesis of SnS_2_ is described in Figure 1. The elevated-temperature synthesis in high-boiling point solvents (oleylamine, oleic acid) was followed by mixed anodic and cathodic electrochemical exfoliation in N-methyl pyrrolidone (NMP). The exfoliation was performed by switching between the anodic/cathodic modii during chronopotentiometric cycling (Figure 1B). The characterization of the exfoliated flakes was performed by Raman spectroscopy, XPS, XRD, and electron microscopy (for TEM see Figure 1C; for EDX/SAED see Figure 1D). Reasons for choosing each of the techniques are explained in the following.

Figure 1A shows the pathway towards synthesis of bulk 2H-SnS_2_ and, as a result, of further electrochemical exfoliation to sheets of SnS_2_. First, the bulk material was synthesized in high-boiling point solvents oleylamine (364°C) and oleic acid (195°C) in 1V:1V volume ratio. In this step, the polymeric structure of tin chloride used as a precursor is end-capped with the amino group of oleylamine, leading to the generation of an iminium radical cation due to the electron transfer to Sn(II)chloride, in a similar fashion as described elsewhere (Radtke and Ignaszak, 2016). The full mechanism can be found in the Supplementary Figure S1. As suggested previously, the electron transfer will reduce tin to Sn(I) and the iminium radical cation will dimerize with oleylamine (Radtke and Ignaszak, 2016) (MourdikoudisLiz-Marzán and Liz-Marzan, 2013). The role of oleic acid is to stabilize the pH of the reaction and to allow the boiling at higher temperatures needed to achieve thermodynamic stability of the pure 2H-phase (Thangaraju et al., 2020).

Following the synthesis of the bulk material, the powder was pressed into a pellet (10 tons) and then electrochemically exfoliated to sheets. The exfoliation was performed by using a Pt wire as counter electrode and the 2H-SnS_2_ pellet hooked on another Pt wire. XPS analysis of the sample revealed no Pt residues on the surface.

Figure 1B shows the chronopotentiometry performed in 10 s cycles by alternating the ± 10 V pulses (single square wave), where the positive part of the square wave is understood as anodic (oxidation), while the negative potential part as cathodic (reduction). Already after 10 cycles the previously in-dispersible 2H SnS_2_ material started transferring into the organic phase of N-methyl pyrrolidone. A TEM image after centrifugation and washing of SnS_2_ with n-hexane is depicted in Figure 1C, showing well-defined flakes of 2D material, which were investigated for their purity by EDX (see below).

The SAED in Figure 1D shows a typical pattern for Sn(IV)S_2_, by comparison with the literature (Hwang et al., 2018). Starting from the inner ring, the crystallographic orientations of (001), (100), (101), (102), (111) and (102) were observed, indicating the presence of the 2H-structure of the P3m1 space group (Hwang et al., 2018). The presence of these reflexes is further confirmed by XRD analysis (Supplementary Figure S3).

EDX analysis confirms the purity of the sample (Cu lines arise from the support TEM-grid), while XRD analysis of the 2D SnS_2_ material reveals a characteristic 2θ peak a 15.02° pointing to the (001) phase (Supplementary Figure S3). After Rietveld analysis, the presence of the 2H-phase was confirmed by reflexes at 28.25° (100), 32.15° (101), 41.98° (102), 50.12° (110), 52.45° (111), and 60.62° (201), which is in good agreement with the literature (Ou et al., 2015). Coherence of the Raman measurements also do not indicate any presence of additional species (Supplementary Figure S4).

XPS analysis based on the Sn3d photoemission revealed two major peaks at 495.0 (Sn3d_3/2_) and 486.6 eV (Sn3d_5/2_) with a coupling constant of 8.4 eV (Supplementary Figure S5), which corresponds to the Sn(IV) oxidation state (Whittle et al., 2016). The lack of oxide within the XP spectra indicates that the air-free technique of synthesis was successful, while residues of the oleic-acid (the structure C_18_H_34_O_2_ indicates the C1s:O1s ratio to be 9:1), possibly van der Waals end-capped onto the tin, contribute to the considerable C1s and O1s signals (70.5% C1s and 7.1% O1s yielding a ratio of 9.9:1), while the remaining carbon contribution is attributed to adventitious carbon. Please note that no oleic acid and SnO_2_ residues were observed in the Raman spectra. The absence of SnO_2_-related features in the Raman spectra corroborates that the oxide is confined to the surface.

A peculiarity of the XPS analysis is the Sn/S ratio of nearly 1, which has been reported previously and attributed to the relatively poor S2p cross-section within the structure (Whittle et al., 2016). The results of a detailed XPS analysis with calculated ratios of atomic percentages can be found in Supplementary Table S1. Please note that the XP spectra were acquired post-mortem, which could result in possible interaction with the environment.

Based on this synthesis approach a single 2H-phase was generated, which is characterized by a single A_1g_ mode at 314 cm^−1^ without a low wavenumber side-band, which would indicate other polytypes such as 2T with a Raman feature at 300 cm^−1^ (Figure 2). Similarly, unlike other synthetic approaches, by-products were observed neither in bulk nor after electrochemical exfoliation, as judged by the lack of Raman signals corresponding to 4H and 18R polytypes of SnS_2_ (Pałosz et al., 1990) (Thangaraju et al., 2020). Due to the use of air-free Schlenk techniques, no Raman features indicating the presence of SnO_2_ or monosulfides like π-SnS were observed in the pristine material.

### 3.2 Raman analysis of the exfoliated SnS_2_

In order to evaluate the SnS_2_ material, a detailed vibrational Raman analysis at 633 nm excitation was performed, revealing a prominent A_1g_ band at 316 cm^−1^ and vague E_g_ band at 158 cm^−1^ (Figure 2), which is consistent with the literature (Smith et al., 1977), as well as longitudinal acoustic phonon modes and the E_g_ band (Figure 2A). At 330 cm^−1^, an additional feature is observed after deconvolution of the A_1g_ band at 316 cm^−1^, which is tentatively assigned to a S^2-^ vacancy (Xia et al., 2016).

The irreducible representation within 2D SnS_2_ differs from bulk SnS_2_ due to the removal of the vertical direction and the point group D_3d_ (multilayer) transforms into D_3h_ (monolayer), yielding Γ = A_1g_ + A_2u_ + E_g_ + E_u_, whereby only the gerade „g“-modes are observed in Raman spectra (A_1g_ and E_g_). For SnS_2_, the E_g_ mode is strongly suppressed, contrasting the behavior of other TMDs (Rehmana and Ding, 2018) (Skelton et al., 2017). This behavior has been explained by the octahedral coordination environment favoured by Sn^4+^, as opposed to square-pyramidal coordination within Fe^4+^-, Ti^4+^- or Mo^4+^S_2_ (Domagoj and Wilson, 2020) (Wang et al., 2017).

As shown in Figure 2A, the presence of the expected E_g_ Raman active mode (in-plane stretching vibration of S-Sn-S-) was observed (Yang et al., 2016) (Gurnani et al., 2017). This unique behavior of Raman band intensities (A_1g_ vs. E_g_) within few-layers exfoliated SnS_2_, as compared to other transition-metal-dichalcogenides, can be explained by the tetrahedral symmetry in tin compounds in contrast to the octahedral symmetry in iron, molybdenum or titanium disulfides (Volgmann et al., 2017) (Han and Hu, 2016). A more detailed analysis based on symmetry considerations can be found in the following spectroelectrochemical section.

Prior inclusion of the exfoliated few-layer SnS_2_ into the battery, in order to improve the conductivity, the relatively broad-bandgap 2D SnS_2_ material (2.3 eV) was mixed with graphite, but with exclusion of a binder in order to avoid overlapping of Raman spectra with the signals of poly (vinylidene difluoride) (Radtke and Hess, 2021) (Gajendiran and Rajendran, 2011). In previous studies, after mixing of 2H SnS_2_ with L-cysteine and after subjecting the mixture to hydrothermal reaction, a series of additional Raman features in form of pentatomic and heptatomic rings were observed (Shown et al., 2018). In contrast, simple mechanical mixing of SnS_2_ with carbon (graphite) in a mortar did not lead to characteristic bands for pentatomic and heptatomic rings, which were observed elsewhere (Doyle and Dennison, 1995) (Lin-Vien et al., 1991). In case of our study only one Raman feature at 1635 cm^−1^ was identified during the *operando* study, which arises from the presence of SnS_2_ deposited on graphite (C=C unoxidized sp^2^ stretching vibration) (Deshmukh et al., 2015). On the other hand, we did not observe a C-S feature at 728 cm^−1^ during the cycling, which would indicate a reaction between graphite and sulfur in SnS_2_. For possible interactions with the electrolyte during *operando* conditions leading to the formation of the signal at mentioned 1336 cm^−1^ please see Supplementary Figure S6. Detailed information from the Raman spectra indicate, that the change of oxidation state can occur rapidly (Supplementary Figure S7). A peculiar behavior is also observed in generation of sulfur vacancies (Supplementary Figure S8).

The A_1g_ mode arises from the translational interlayer phonon S-Sn-S and the E_g_ from the longitudinal stretching vibration (Smith et al., 1977) (Zhang et al., 2018). As it was reported by others, the chalcogenides and dichalcogenides tend to bond with alkali metals, especially during electrochemical perturbation (Leng et al., 2016). As the monolayers of chalcogenides are known for not having dangling bonds on the surface, the chemical intercalation may be expected to occur *via* bond generation between sulfur and alkali metal. One may therefore expect both modes to be affected by intercalation. In this work, we focused on the translational A_1g_ mode, which is not blurred by the presence of the electrolyte (MPF6/PC, M = Li^+^) (Radtke and Hess, 2021).

### 3.3 Electrochemical characterization and evaluation of 2-dimensional SnS_2_

**FIGURE 3:**
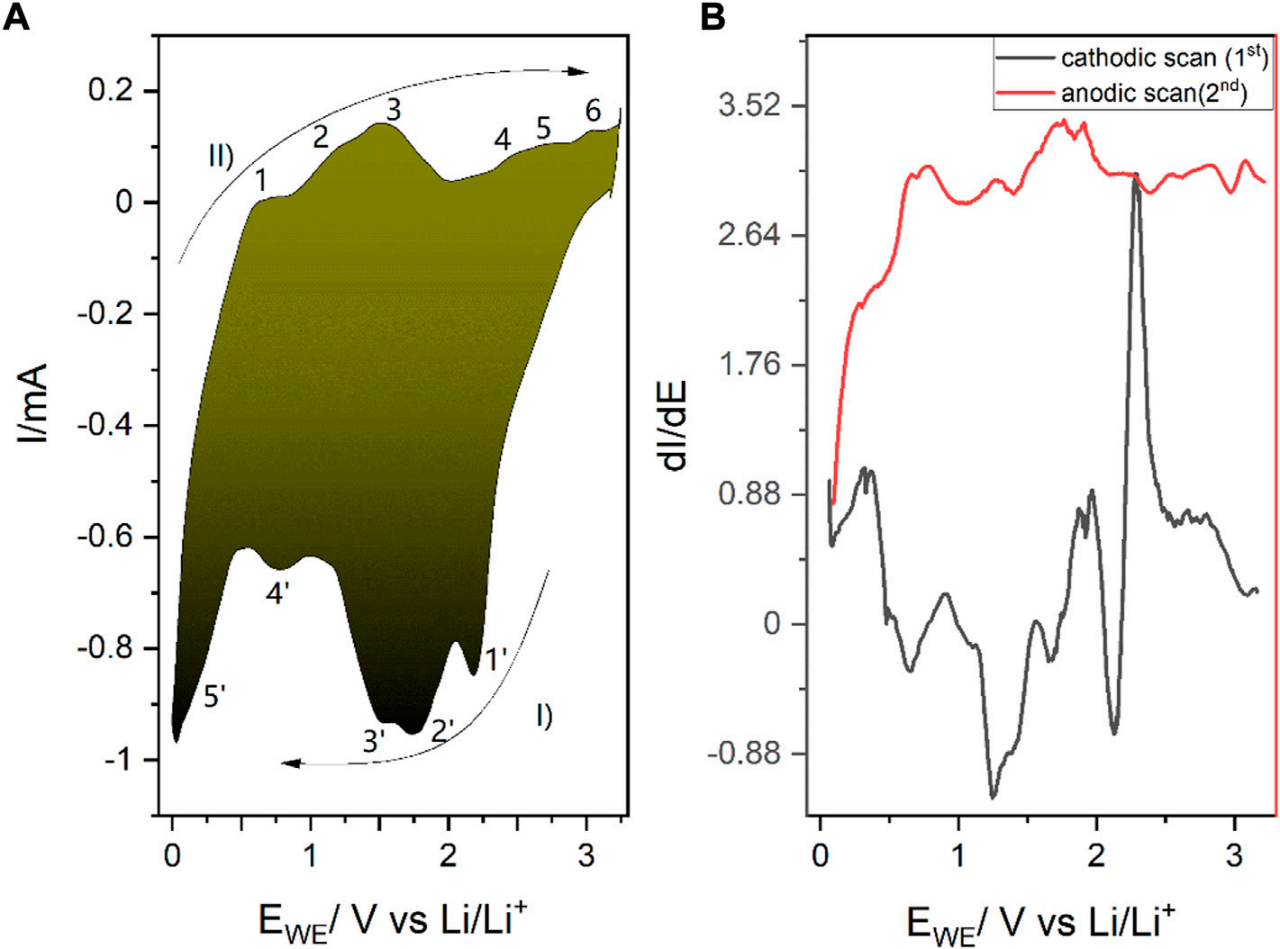
**(A)** Electrochemical cyclic voltammetry scans of the 2D SnS_2_ with 0.01 mV/s scan rate during Li^+^ (de)intercalation/alloying. I) refers to the forward and II) to the reverse scan, while the consecutive numbers correspond to the reactions listed in Table 1. **(B)** Corresponding dI/dE plot for a more pronounced representation of the electrochemical peaks. The observed transitions are gathered in Table 1 and discussed in the text. The cathodic scan was performed prior to the anodic scan.

**FIGURE 4:**
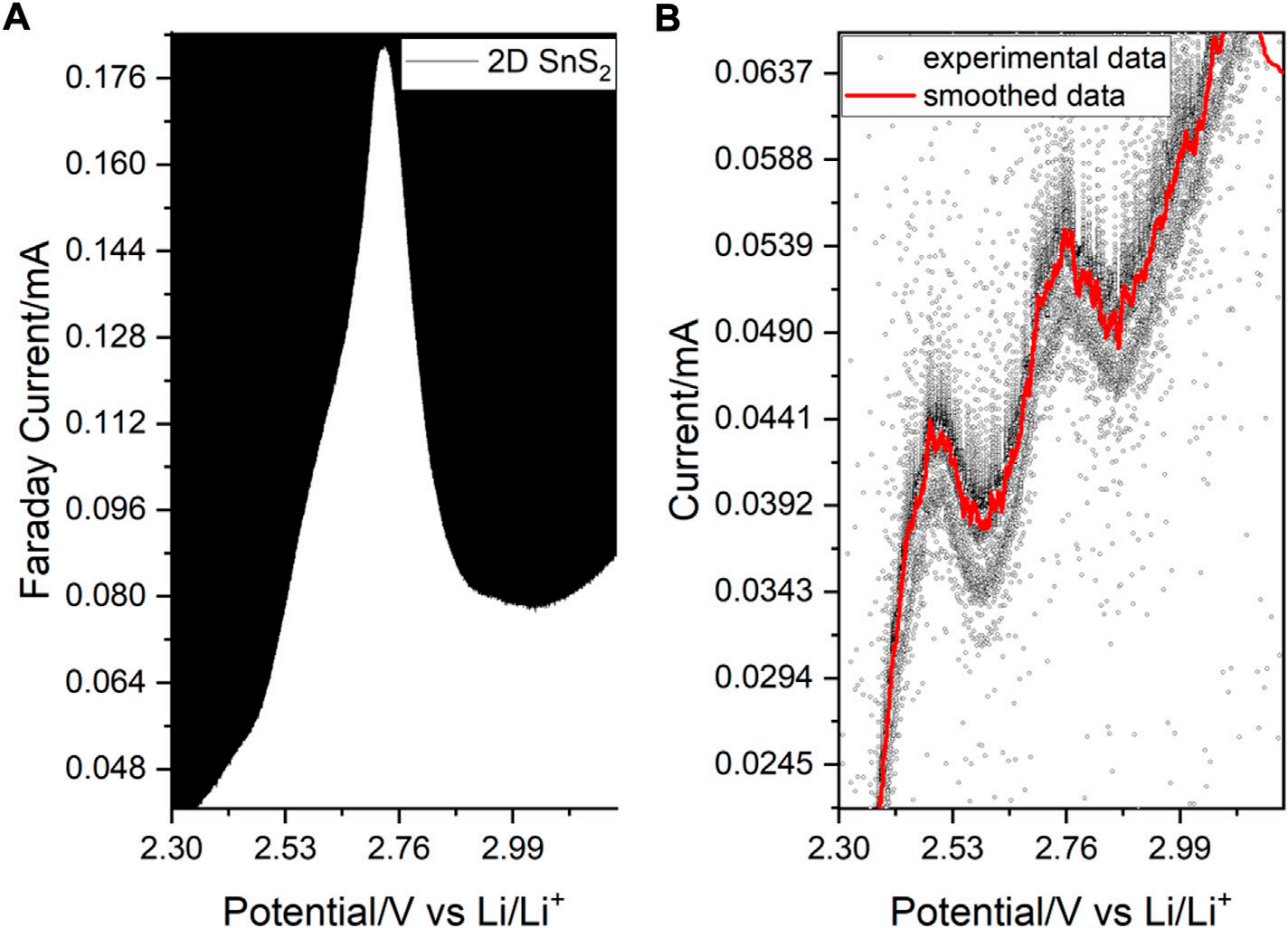
**(A)** Anodic DPV spectrum showing a major peak with a hidden contribution. **(B)** SPEIS-deconvoluted current/potential relationship involving two peaks grasped during 1399 potential steps (37 frequency points per step), which are seen in A as a shoulder preceding the main peak.

Prior to the *operando* experiments, the 2D SnS_2_ was thoroughly analyzed by electrochemical means. Figure 3A shows the 2-electrode system CV of the 2D SnS_2_ incorporated into the lithium-ion-battery, whereas Figure 3B shows the dI/dE derivative spectrum of the CV acquired in order to better elucidate the observed peaks. Table 1 contains a description of the major peaks observed during the scan, which are furthermore propagated by more advanced analysis in Figure 4 (DPV and a quasi-CV-approach obtained from impedance linearization).

**TABLE 1:** Reactions taking place during the spectroelectrochemical scan of 2D SnS_2_ with the assigned potentials during Li^+^ (de)intercalation/alloying. The numbers refer to Figure 3.

Anodic scan reactions and potentials in V	Cathodic scan reactions and potentials in V
1 0.6 Li_x_Sn → xLi^+^ + Sn + xe^−^ (Hwang et al., 2018)	5′ 0.4 Sn + 4.4Li^+^ + 4.4e^−^ → Li_4.4_Sn (Kisu et al., 2014)
2 1.24 Li_4.4_Sn → Sn + 4.4Li^+^ + 4.4e^−^ (Kisu et al., 2014)	4′ 0.76 Li_x_SnS_2_ → SnS_2_ + xe^−^ (Mahmud et al., 2022)
3 1.54 Sn + 2Li_2_S → Li_x_SnS_2_ + (4-x) + 4e^−^	3′ 1.52 Li_x_SnS_2_ +(4-x)Li^+^ → Sn + 2Li_2_S (Hwang et al., 2018)
4 2.19 SnS_2_ + xLi^+^ + xe^−^ → Li_x_SnS_2_ (Kisu et al., 2014)	2′ 1.75 SnS_2_ + xLi^+^ xe^−^ → Li_x_SnS_2_ (Mahmud et al., 2022)
5 2.49/2.67 SnS_2_ + xLi^+^ + xe^−^ → Li_x_SnS_2_/generation of sulfur chains -S-S-S-/tin disproportionation (Zhou et al., 2017)	1′ 2.17 Li_x_SnS_2_ + Li_2_S → SnS + S + (2+x)Li^+^ (Chen et al., 2016)
6 3.04 generation of sulfur chains and generation of SnS_2_-Li bond alongside further sulfur chains (Zhou et al., 2017)	

All spectra in Figure 3 were acquired with the cathodic scan being the forward direction and the anodic the reverse. The observed transitions are summarized in Table 1. As the intercalation onto the available sites occurs in a reductive way, the cathodic scan was performed first (Massé et al., 2017).

Figure 3A is characterized by five peaks in the forward cathodic scan and six major anodic peaks in the reverse scan. The numbers by the peaks correspond to the numbers in Table 1. Nevertheless, detailed analysis of the dI/dE = f (E vs. Li/Li^+^) reveals 12 pairs of peaks. As Table 1 shows, some reactions of alloying and intercalation have an effect on current generation, despite of not being purely of RedOx Faraday character. For instance, in the region between 1.52 and 1.75V vs. Li/Li^+^ in the anodic scan (reactions 3′ and 2’), an unexpected intercalation occurs due to the chemical reaction between SnS_2_ and positively charged lithium ions. Interestingly, in case of 2D SnS_2_ we have observed the generation of Li_2_S not to be deterministic for cycling, as it was the case for the bulk material.

Intercalation of the 2D SnS_2_ occurs in similar fashion to the 3D material, i.e. within 0–1 V vs. M/M^+^. In case of Li^+^ intercalation, the occurring alloying reaction is visible at 0.4 V, where the reaction 0.4 Sn + 4.4Li^+^ + 4.4e^−^ → Li_4.4_Sn takes place primary in the cathodic scan (Kisu et al., 2014). Interestingly, some Faradaic reactions responsible for the intercalation also occur in the anodic scan, with 1.54 and 2.19 being the most prominent examples. Reasons underlying this behavior do not come from the pure RedOx mechanism, but rather from associative reactions.

Interestingly, instead of the usually final step involving Li_2_S generation, Li_2_S further reacts with 2D SnS_2_ in the anodic scan as Sn + Li_2_S → Li_x_SnS_2_ + (4-x)e^−^ (1.54 V) (Hwang et al., 2018). The previous reaction (cathodic scan was performed first) at 2.19 V in the anodic scan is another example of intercalation due to the reaction SnS_2_ + xLi^+^ → Li_x_SnS_2_ (Mahmud et al., 2022).

To this end, we performed additional measurements, which revealed the (III) oxidation state of tin, as described in the following. In the CV in Figure 3A, as well as in Figure 4, the reactions at 2.19 and 2.49 V are of particular interest, as they encompass the electrochemically grasped creation of Sn(III), as previously postulated for “very thin materials” (Chang and Bard, 2014).

During the anodic scan the expected oxidation is overshadowed by the prevalent tin disproportionation, where species at both the highly unusual, but still previously predicted (III) and the casual 4th oxidation state occur, ending on the creation of tin (II)sulfide at 3.04 V vs. Li/Li^+^. Tin disproportionation was reported previously in case of Sn(II) within tin (II)-2,3-dihydroxysuccinate (tin tartrate reacting with itself): 2Sn^2+^ → Sn^4+^ + Sn(0) (Cartwright and Woolf, 1977) (Chapman, 1913). Note that the positions marked as 5’/6’ are the starting point for 1, where the anodic processes of Li_x_SnS_2_ start to take place.

In anodic DPV, the RedOx activity of 2D SnS_2_ was observed at 2.55 V vs. Li/Li^+^, which arises from the Sn-S activity and a mixed phase rearrangement/oxidation state change at sulfur, which leads to Li^+^-ion accommodation (Yanyun et al., 2020) (Yang et al., 2020). The main peak exhibits a strong asymmetry, which corresponds to the Sn-S phase transformation at 2.53 V and generation of sulfur chains with lithium at 2.76 V (Xiao et al., 2017).

The two peaks were resolved upon SPEIS-deconvolution (1399 potential steps for increased sensitivity during the AC-voltammetry scan involved in frequency-dependent SPEIS) of the current/potential relationship during the impedance scan (Figure 4B). The DPV scan was additionally used to evaluate the separation of the HOMO/LUMO positions within graphite mixed with 2D SnS_2_, which was established to be 1.69 eV during the intercalation at 1.75 vs. Li/Li^+^, explaining the good material conductivity (for details see Supplementary Information). In this context we note that the SnS_2_ anode consisted of no additional conductive carbon nor any binder (e.g. PVDF), which may interfere with the Raman signal (Deepa and Nagaraju, 2012). The sharp transitions observed in Figure 4 are to be related with the 2-dimensional character of the structure. Considerably large ionic and electron mobilities of the 2D SnS_2_ are evaluated in the following subsections.

### 3.4 *Operando* Raman spectroelectrochemical investigation of lithiation of 2-dimensional SnS_2_

**FIGURE 5:**
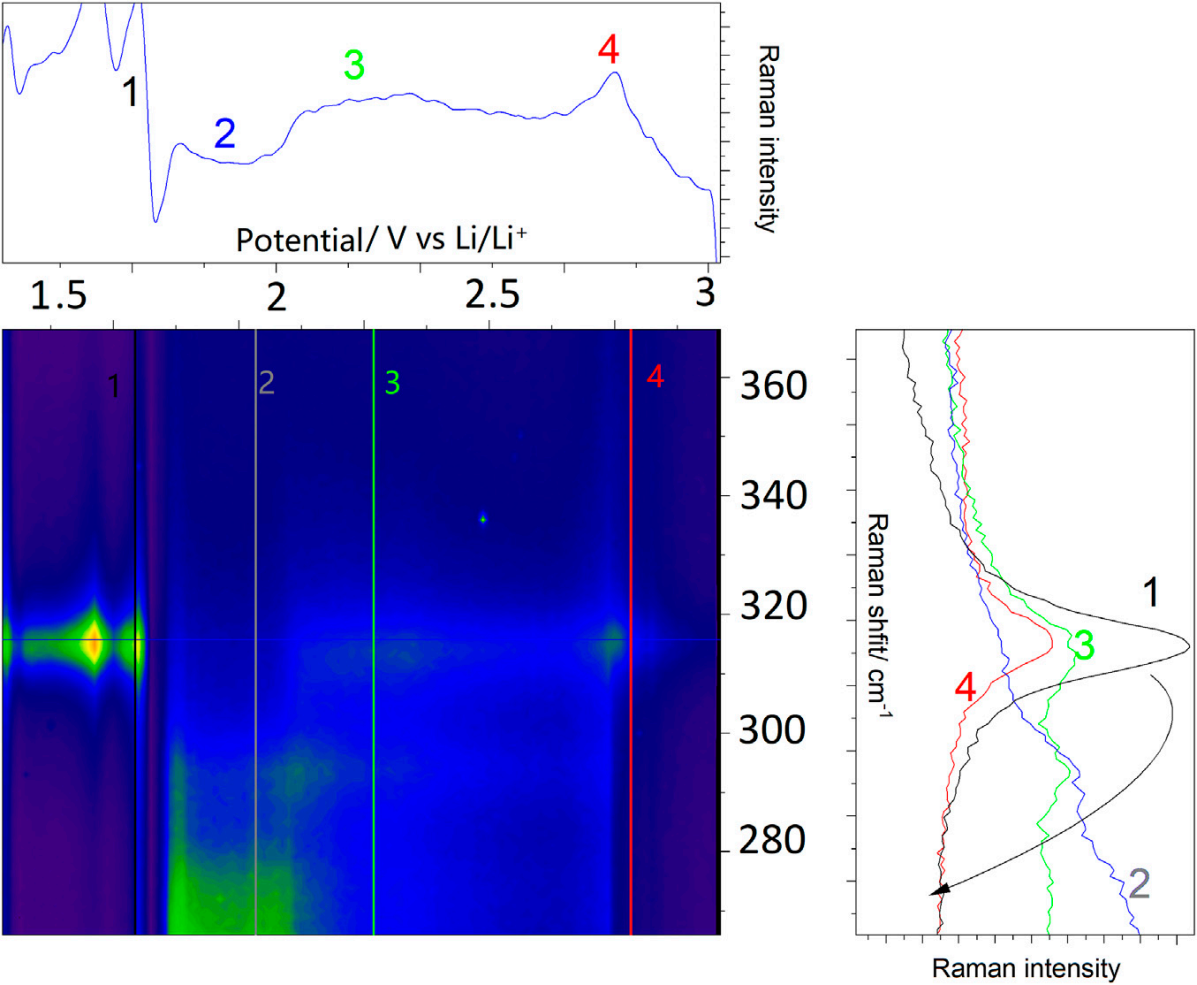
*Operando* Raman spectroelectrochemistry during lithiation of 2D SnS_2_. The right panel shows consecutive steps during 2D SnS_2_ lithiation. Colored numbers refer to the potentials, at which the Raman spectra were acquired and correspond to the vertical lines in the central contour. The intensity changes at 1.65 V result from an intermediary loss/recovery of laser focus. For details see text.

Following the characterization, *operando* experiments were performed, where structural changes within SnS_2_ during lithiation were monitored by vibrational Raman spectroelectrochemistry, with a focus on the most prominent A_1g_ mode arising from the out-of-plane S-Sn-S- stretching vibration, which was strongly affected by the presence of lithium, sodium and potassium at higher potentials (Gonzales and Oleynik, 2016). Figure 5 depicts *operando* Raman spectra, which were acquired simultaneously to a DPV experiment as shown in Figure 4. In a specially designed spectroelectrochemical cell at each potential with 0.09 mV precision a Raman spectrum was acquired, which upon accumulation of spectra resulted in the contour plot shown in Figure 5. Position 3 at 2.26 V shows a bimodal A_1g_ distribution, arising from the degeneracy imposed by both electrochemical potential and generation of the Sn-S-Li bond (see below), further described as A_1g_ and A_1g_’. Position 4 shows a slightly blue-shifted A_1g_ mode (with regard to position 1 prior to the reaction), which results from the accommodation of Li^+^-ions in the SnS_2_ structure (Xiong et al., 2017).

**FIGURE 6:**
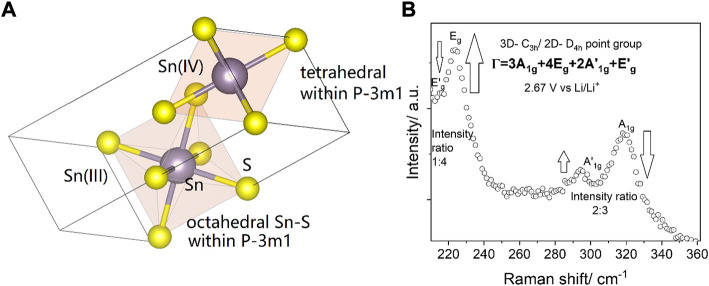
**(A)** The double occurrence of A_1g_ and E_g_ Raman active modes is proposed to arise from C_3h_ and D_4h_ symmetries within the 2H polytype of SnS_2_, which belongs to the P-3m1 class. **(B)** In the *operando* Raman spectrum of the 2D material at 2.67 V vs. Li/Li^+^, the E_g_ band can be higher in intensity than the A_1g_ band only in octahedral environment, as it was observed for TMDs with pure octahedral symmetry. Sn(IV) favors tetrahedral symmetry (Gurnani et al., 2017). In tetrahedral symmetry A_1g_ prevails over other modes. Octahedral symmetry is only possible in Sn(III) environment (Fitzsimmons, 1968) (Beletskiy et al., 2014). On purpose non-background corrected data are shown. For clear prevalence of the modes after subtraction see Supplementary Figure S7.

**FIGURE 7:**
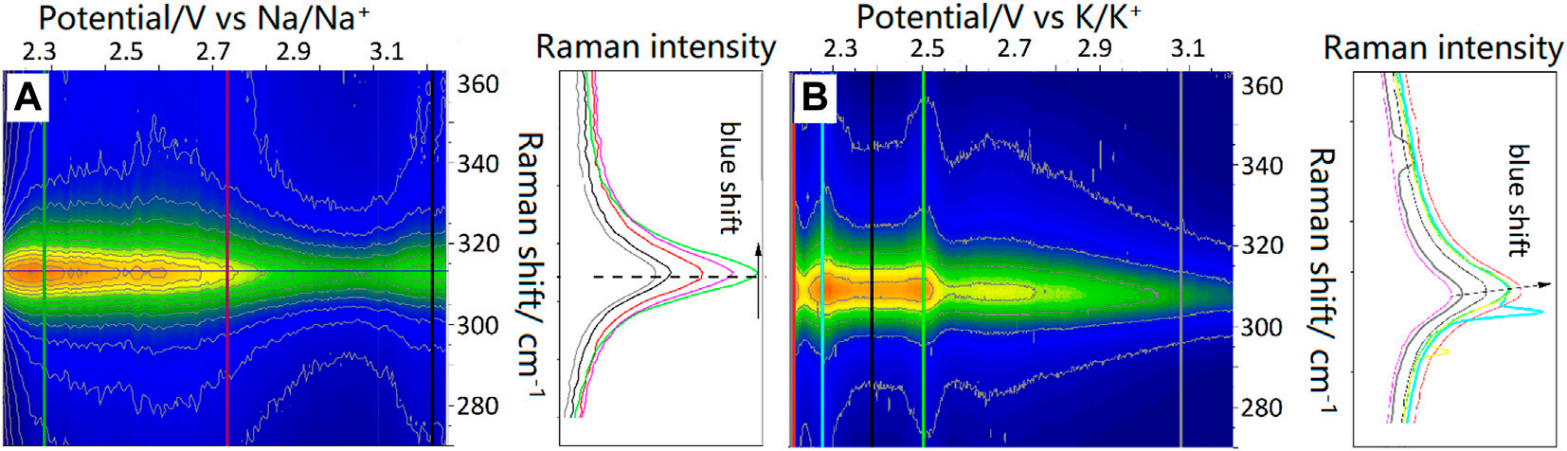
*Operando* spectroelectrochemistry during **(A)** sodiation and **(B)** potassiation of 2D SnS_2_.

As judged by the blue-shift, we do not relate this process to an increase in crystallinity, but rather the accommodation of single SnS_2_ sheets with intercalated ions, which leads to a significant loss of the amorphous character observed in pure 2D-SnS_2_. The pathway from the single A_1g_ mode, through the bimodal distribution and comeback to the single blue-shifted mode (as indicated by the black arrow in the right panel) lays the foundation for the mechanistic analysis in the following subsection (see Figures 6, 7 for the sodiation/potassiation analysis alongside with the Raman proof of the Sn(III) oxidation state). The characteristic SnO_2_ Raman active band at 472 cm^−1^ was observed only shortly below 1.5 V vs. Li/Li^+^ during the *operando* measurement.

The lack of structural breathing in the 2D material causes the Li_2_S not to be the judgmental final step in the cycling stability within lithium-sulfur batteries, as it is the case for 3D SnS_2_ (Vinzentin et al., 2017). The lack of volume expansion in 2D materials has been discussed previously and seems to be confirmed here by the FWHM data of Raman active modes. After normalization of the FWHM data, we have observed no breathing upon sodiation and potassiation, which was postulated as an expected phenomenon from 2D SnS_2_ materials (Pomerantseva and Gogotsi, 2017). This can be seen in the Figure 7, where colored lines do not significantly change in width in the intercalation regions indicated in the Table 1.

In 3D materials the intermediate (III)-oxidation state of LiSnS_2_ was not observed by Raman spectroscopy. An intermediate state of Sn(IV) and Sn(III) can occur due to the creation of a sulfur-lithium bond, whereby the highly electronegative (EN = 2.58) and therefore polarizable sulfur atoms are suggested to be responsible for the pronounced Raman signature. On contrary to the alloyed Li_x_SnS_2_, the LiSnS_2_ state is higher blue-shifted (A_1g_ mode in the 321 cm^−1^ position) with respect to 318 cm^−1^ (Julien and Perez-Vincente, 1996). Figure 6 shows the Raman analysis of the grasped Sn(III) oxidation state.

Despite the symmetry induced presence of A_1g_ mode, during the spectroelectrochemical scan at 2.67 V vs. Li/Li^+^ we have nevertheless observed a typical 2D signature of TMD, where E_g_ and A_1g_ modes are inversed in their intensity (Zhang et al., 2015). The E_g_ mode is of much higher intensity and dominates only at this point over the A_1g_ mode. The observed behavior is at first surprising, as even if this would be expected from other 2D TMDs like FeS_2_ or MoS_2_, the symmetry group of SnS_2_ differs from other transition metal disulfides, but may nevertheless be explained by the presence of a Sn(III) oxidation state with different chemical environment. The presence of Sn(III) was already discussed in the context of SnBr_6_ disproportionation experiments studied by fast-scan CV (Chang and Bard, 2014). The symmetry of Sn(III) is similar to Mo(IV) and therefore we postulate that an intensity inversion is possible (Voiry et al., 2015). Supplementary Figure S7 shows the detailed Raman spectra observed at this potential, i.e. 2.67 V vs. Li/Li^+^. To our best knowledge, the *in-situ* generation of Sn(III) has not been observed previously by spectroelectrochemistry. Supplementary Figure S8 describes the presence of sulfur vacancies.

The Raman spectra were additionally used to monitor the quality of SnS_2_ based on the shape of the A_1g_ mode. Directly after the synthesis only a small amount of sulfur vacancies (S^2−^) was found (5% of the integrated Raman band), which increased drastically upon anodic charging of the material (Supplementary Figure S6). Similar *operando* Raman measurements were also performed during sodiation and potassiation, whereby we have not observed such drastic changes as in case of LIB. The reason for this behavior might be related to the different solvation Stokes-radii of alkali metals with 1M LiPF_6_ in propylene carbonate (PC), which were established to decrease with increasing atomic number and atomic radius (Masese and Kanyolo, 2022). The smaller solvated structures lead to less distortion within the channel-driven mass transport mechanism, as indicated for 2D materials (Lukatskaya et al., 2016). The different mass transport mechanism therefore leads to a lack of volume expansion and thus more stable batteries. Details of the *operando* characterization during sodiation and potassiation will be discussed in the following section.

### 3.5 Sodiation and potassiation kinetics

Figure 7 depicts the *operando* results for the sodiation and potassiation of 2D SnS_2_.

**FIGURE 8:**
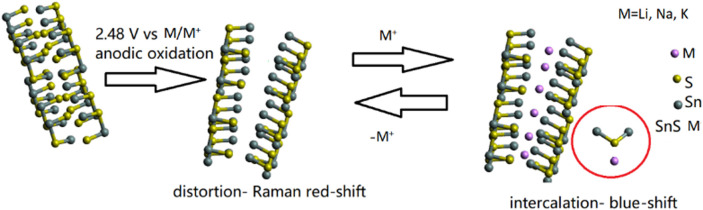
Proposed mechanism of SnS_2_ lithiation consisting of electrochemical lattice distortion monitored by *operando* vibrational Raman spectroelectrochemistry, weak ionic lithium accommodation on the sulfur side and deducted from that blue-shift of the translational S-Sn-S mode in the Raman spectrum.

As it was the case for lithiation, a distortion of the A_1g_ Raman mode is detected. For sodiation there is no visible Raman mode splitting into A_1g_ and A_1g_’ and the observed blue-shift occurs only within 2 cm^−1^. The presence of the blue-shift is peculiar, as for lattice distortion due to metalation rather a red-shift would be expected, as indicated by the proposed mechanism (Figure 8). Based on post-mortem XPS analysis of the sodiated sample, we propose the generation of Sn-S^-^ M^+^ (M = Li, Na, K) bonds to cause the blue-shift. The structure gains crystallinity due to the creation of an alloy rather than intercalation of ions. The alloying of sodium and potassium during electrochemical cycling has recently been proposed for CeO_2_ as an anode (Yanjiao et al., 2020). Such a behavior underpins the application of SnS_2_ as an anode, due to the reduced mechanistic expansion as compared to Si (up to 400% expansion and mechanistic destruction of the anode), but also due to difficulties of inserting sodium and potassium into graphite (Ellis et al., 2014).

As indicated by Table 2, the sodiation occurs almost undistorted as suggested by the charge transfer coefficients for the anodic (0.52) and cathodic (0.48) processes from the Butler-Volmer analysis (for more details see the Supplementary Information). Nevertheless, the right panel of Figure 7 may provide insight into the structural evolution during sodiation and potassiation based on changes of the FWHM (virtually no changes). After closer inspection of the period from 2.3 to 3.0 V vs. Na/Na^+^ in NIB there is a FWHM decreaseof 7 cm^−1^, which does not change afterwards. This was not observed for KIB.

**TABLE 2:** Changes within the A_1g_ Raman active mode at potentials indicated in Figure 3, diffusion coefficients extracted from the PITT, electron mobility extracted from the SPEIS measurement and kinetic merits of lithiation based on Butler-Volmer analysis. Corresponding Figures were gathered in the Supplementary Information (Supplementary Figures S9–S19).

Electrochemical data	Potential/V^a^	A_1g_ mode Li^+^	A_1g_ mode Na^+^	A_1g_ mode K^+^
*Operando* Raman spectroelectrochemistry^b^	1.80	316 cm^−1^	316 cm^−1^	316 cm^−1^
	2.55	298 cm^−1^	317 cm^−1^	318 cm^−1^
	3.11	320 cm^−1^	317 cm^−1^	319 cm^−1^
Charge transfer coefficient^c^	Cathodic	0.32	0.48	0.21
	Anodic	0.68	0.52	0.79
Diffusion coefficient^d^ cm^2^s^−1^	Before the reaction	4.57E-07	8.11E-08	2.75E-08
	During the reaction	2.75E-07	4.45E-08	4.57E-08
	After the reaction	5.15E-07	1.03E-08	8.02E-09
Electron mobility^e^ cm^−1^Vs^−1^	Before the reaction^f^	1.79E-02	2.42E-05	9.18E-03
	During the reaction^f^	2.28E-02	2.54E-05	1.59E-02
	After the reaction^f^	2.96E-02	2.68E-05	1.19E-01

^a^ vs. Li/Li^+^, Na/Na^+^, K/K^+^, respectively.

^b^ 633 nm Raman probing of the 2D SnS_2_ material every 18 mV.

^c^ Established from Tafel polarization experiments (Figure 5, inset A).

^d^ Established from the PITT experiment.

^e^ Obtained from the equivalent circuit modelling (Figure 5, insets C and D).

^f^ Prior to the reaction: 1.8V; during the reaction: 2.72V; after the reaction: 3.51 V.

One possible explanation of this effect may arise from surface sulfur in 2D-SnS_2_ reacting with alkali metals. As DFT studies of Li^+^ insertion into CeO_2_ have shown that surface oxygen plays a crucial role in intercalation, we postulate that in case of lithiation/sodiation/potassiation of SnS_2_ a similar process may takes place with sulfur (Cortes et al., 2020). The blue-shift seen in Figures 7A,B has been attributed to the strain induced by the van der Waals interactions between the stacked few-layers 2D SnS_2_ (Sriv et al., 2018).

In case of sodiation there is a blue-shift, which is irreversible, but in contrast to lithiation and potassiation, the Raman intensity after 3.0 V vs. Na/Na^+^ increases. While the Raman intensity may be influenced by several factors, intercalation of sodium into SnS_2_ may generate a structure, which is in resonance with the 633 nm laser excitation red laser, thus leading to an increased Raman cross section. In case of potassiation there are three main distinctive peaks observed in the Raman intensity as a function of the applied potential. The first occurs at 2.3 V vs. K/K^+^, the second at 2.6 V vs. K/K^+^, and the third as a depressed band between 2.7 and 2.9 V vs. K/K^+^. As it was the case for sodium, there is a continuous blue-shift upon potassiation, also indicating the generation of Sn-S-K bonds. There is nevertheless also a pronounced multiple Raman active mode splitting at potentials matching the broadening of the FWHM (2.3 and 2.6 V vs. K/K^+^), as based on the normalized spectra.

As in case of lithiation, this splitting may occur due to the generation of the additional bonds between the layers, which are being first distorted by intercalation and then chemically changed. The kinetic studies of these processes indicate, that they happen faster than in sodium, but slower than within LIB (Table 2). The blue-shift in Raman amounts to 3 cm^−1^.

The diffusion coefficient for LIB was calculated to be 4.57E-07 cm^2^s^−1^ before the reaction (1.8V). During the RedOx reaction (2.55 V) and lithium accumulation, the diffusion coefficient dropped to 2.75E-07 cm^2^s^−1^ as expected, while after the saturation, the lithium movement was the highest and can be contributed mostly to the electrolyte movement (Warburg impedance depicted as „W“ in inset C of Supplementary Figure S20), which could be observed by the increased diffusion coefficient 5.15E-07 cm^2^s^−1^. Changes in diffusion coefficients indicate a dynamic system where the metalation reactions occur.

A similar trend should be expected from the electron mobility, as the lithiated compounds should exhibit a decreased bandgap and therefore faster transition times of the electrons (Liu et al., 2013). The electron transition was evaluated by means of SPEIS equivalent circuit modelling (model shown in Supplementary Figure S20C); the spectral validation by Kramers-Kronig relations is shown in panel D (3% error), which speaks for both the validity of the scan itself and the good choice of the equivalent circuit model (You et al., 2020).

Calculated electrochemical merits were gathered in Table 2. Prior to the reaction SnS_2_ + xLi^+^ → Li_x_SnS_2_ indicated in Table 1 (1.80 V) the electron transition was calculated as 1.79E-02 Vs^−1^cm^−1^, during the reaction (2.55 V) as 2.28E-02 Vs^−1^cm^−1^, and after the reaction as 2.96E-02 Vs^−1^cm^−1^. These processes were the slowest in case of sodiation and second fastest in case of potassiation. This is most probably caused by the fact, that in case of sulfur interaction with sodium and potassium, this effect is more pronounced than with lithium due to the higher ionization energy of Na^+^ and K^+^ ions when compared with lithium.

The observed behavior may be explained by the solvation radius (with PC), which tends to decrease with higher atomic radius. As the solvated alkali metal ions are responsible for the alloying/intercalation events, the smaller Stokes radii lead to faster kinetic events (Wootton and Zink, 1997) (Hosaka et al., 2020). The events have to be compensated by the strength of bond creation though, which reveals itself in the mismatching diffusion coefficients of potassium/sodium and lithium movements (Table 2). Table 2 summarizes the calculated electrochemical merits. The peculiar behavior of potassiated 2D SnS_2_ after the reaction (faster electron mobility) is not fully understood yet.

As the diffusion coefficients were found to be smallest during the RedOx reaction and electron transition times were increasingly rising with the potential, the lithium accommodation is expected to lower the HOMO/LUMO separation of the SnS_2_ material, which was preliminarily already established as small (1.69 eV at reaction maximum) during the first scan. The lowered HOMO/LUMO separation speaks for an enhanced electron mobility, while the lowered diffusion coefficient at the reaction maximum indicates ion accommodation between the SnS_2_ layers. The diffusion coefficient then rises again at higher potentials, which corresponds to the saturation of layers with lithium ions making them even harder to accommodate at higher potentials. SPEIS studies in Supplementary Figures S9–S20 confirm this hypothesis, as the ionic conductivity region governed by the Warburg impedance is highest at above 3.74 V vs. Li/Li^+^.

Raman and TEM microscopic analysis combined with EDX as well as XPS and XRD confirmed the presence of the desired structure, chemical purity and stoichiometry of 2D SnS_2_. For further TEM images please see Supplementary Figure S21. Supplementary Figure S22 shows the creation of the 3^rd^ oxidation state, while Supplementary Figure S23 shows cycling stability of the lithium/sodium and potassium ion batteries. The *operando* Raman spectroelectrochemical experiment during lithiation indicates first a considerable red-shift during the Faradaic reactions indicated in Table 1, which is attributed to lattice distortion, while the comeback to the slightly blue-shifted A_1g_ position indicates the formation of a lithiated compound (Sole et al., 2014). In the potential region of the RedOx reaction, the Raman spectrum is characterized by a bimodal distribution of the A_1g_ mode, which might occur due to the generation of Sn-S^-^ Li^+^ bonds. As the SPEIS current/potential relationship indicates RedOx activity and oxidation of the sulfur atom, this hypothesis of S-M bond creation was investigated further by means of XPS. XPS has confirmed the shift towards oxidized tin binding energies, which we relate to the pronounced interaction. The Sn/Li (Na, K) ratio was established by post-mortem XPS analysis of the lithiated SnS_2_, the results of which are gathered in Supplementary Table S1. The analysis of point defects can be found in Supplementary Table S2.

## 4 Conclusion

In the presented study we have developed an affordable synthetic approach of 2D SnS_2_ by combined wet-chemical synthesis followed by electrochemical anodic exfoliation. After the characterization of the obtained structure by means of electron microscopic (BFTEM, SAED) and spectroscopic (XPS, EDX, Raman spectroscopy) analysis, we performed an *operando* Raman spectroelectrochemical investigation of the lithiation process of 2D SnS_2_, which has revealed an unusual oxidation state (III) of tin. According to our spectra, the most prominent Raman active mode, the S-Sn-S stretching translational A_1g_ mode was the most affected by application of the external potential. The considerable 17.9 cm^−1^ red shift indicates that lattice deformation occurs during the maximum of the RedOx reaction, while the Li^+^ accommodation at the sulfur side of 2D SnS_2_ within its distorted lattice results in improved crystallinity, as indicated by the blue-shift of the Raman A_1g_ mode. In case of sodiation and potassiation blue-shifts were observed, but spectral characteristics did not change so drastically during the course of the reaction. We proposed a microscopic model of the 2D anode operation and established its applicability by means of electrochemical testing (SPEIS, Tafel, PITT, DPV). In conclusion, 2D SnS_2_ can be easily synthesized and applied as a modern anode for high-energy batteries, while our *operando* vibrational analysi contributes to the mechanistic understanding underlying energy storage. Our results suggest the generation of Sn-S-M (M = Li, Na, K) bonds, instead of van der Waals intercalated compounds, speaking well for the applicability of 2D SnS_2_ as an anode material. The reversibility of the alloying reactions introduces an argument for effective intercalation of alkali metals into the anodes.

## Data Availability

Data supporting conclusion can be made available upon reasonable request.

## Abbreviations

BFTEM, bright field transmission electron microscopy; DPV, differential pulse voltammetry; PC, propylene carbonate; PITT, potentiostatic intermittent titration; SALC, symmetry adapted linear combination; SPEIS, staircase potentiostatic electrochemical impedance spectroscopy; TMD, transition metal dichalcogenide.
