# Supplementary material for: Operando spectroelectrochemistry of bulk-exfoliated 2D SnS2 for anodes within alkali metal ion batteries reveals unusual tin (III) states
Source: Front Chem. 2022 Oct 21;10:1038327. doi: 10.3389/fchem.2022.1038327 (PMC9633852; doi:10.3389/fchem.2022.1038327)
Supplement: Supplementary file 1 [file DataSheet1.docx]

Supplementary Material

*Operando* spectroelectrochemistry of bulk-exfoliated 2D SnS_2_ for anodes within alkali metal ion batteries reveals unusual tin(III) states

M. Radtke^*^, C. Hess

Eduard Zintl Institute of Inorganic and Physical Chemistry, Technical University of Darmstadt, Alarich-Weiss Str. 8, 64287 Darmstadt, Germany; [mariusz.radtke@tu-darmstadt.de](mailto:mariusz.radtke@tu-darmstadt.de)

*Correspondence: mariusz.radtke@tu-darmstadt.de


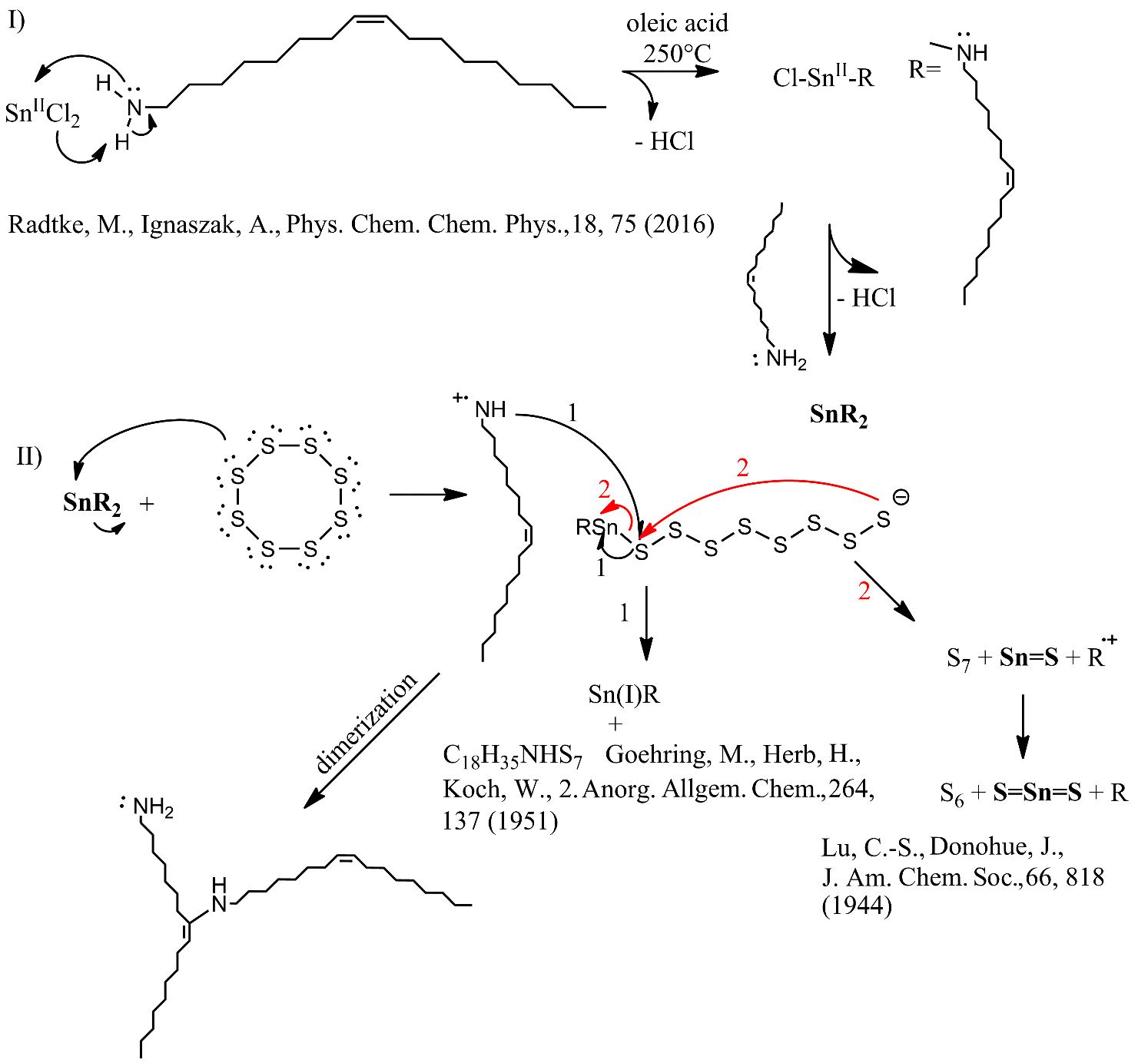


**Supplementary Figure S1**. Possible oleylamine-induced reduction pathway as a 2-step process. I) Reaction of Sn(II)Cl_2_ with C_18_H_35_NH_2_ (oleylamine) upon evolution of hydrochloric acid as gas, which was observed during the reaction. Oleic acid in this reaction serves as a solvent and other than in case of oleylamine does not react with oleic acid to form a sol-gel structure [1]. The step of hydrochloride evolution occurs twice and the end structure is a labile double-capped tin amide core on the second oxidation state with two oleylamine groups connected to Sn by an amide bond. The oxidation state of Sn changes in the second step II. Here the free-electron pair of sulfur reacts with the tin amide upon extraction of the instable oleylamine imine radical cation [1]. The typical cyclic octagonal sulfur cage opens and the oleylamine radical cation is stabilized by the octasulfur anionic chain. Here we propose two possibilities. In first case, the reactive radical cation of oleylamine reacts with the sulfur chain to generate a stable heptagonal sulfur cyclic cage with an amine adduct, also observed for other amine products (S_7_NH) [2]. Here Sn(I) will be generated and this reaction is considered as undesirable. The second possibility is a ring closure (red arrow), the generation of a heptatomic sulfur cyclic cage and the exclusion of the imine radical cation oleylamine from the amide product by generation of tin sulfide (Sn=S). While working with a double of molar equivalent of sulfur, the reaction follows a similar pathway to generate Sn(IV)S_2_, which is depicted in the Figure as reaction with S_7_ upon exclusion of the S_6_ byproduct. Centrifugation and washing of the products with dry n-hexane eliminates the impurities and leaves non-oxidized SnS_2_ as a bulk.


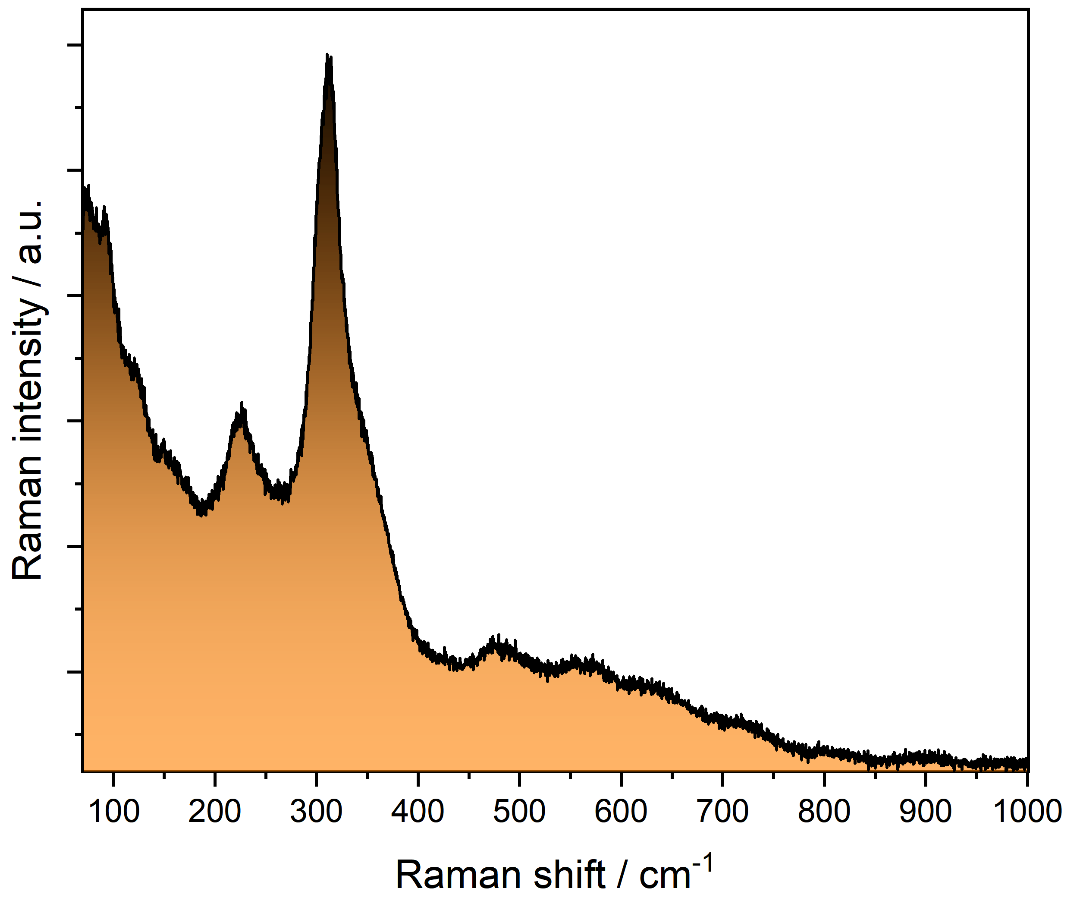


**Supplementary Figure S2.** Raman spectrum (633 nm) of the exfoliated SnS_2_, matching the literature [5]. Please note the low position of the longitudinal acoustic phonons below 200 cm^-1^, which are located much lower than the modes observed during exfoliation in e.g. Figure S19. For more details, see main text.


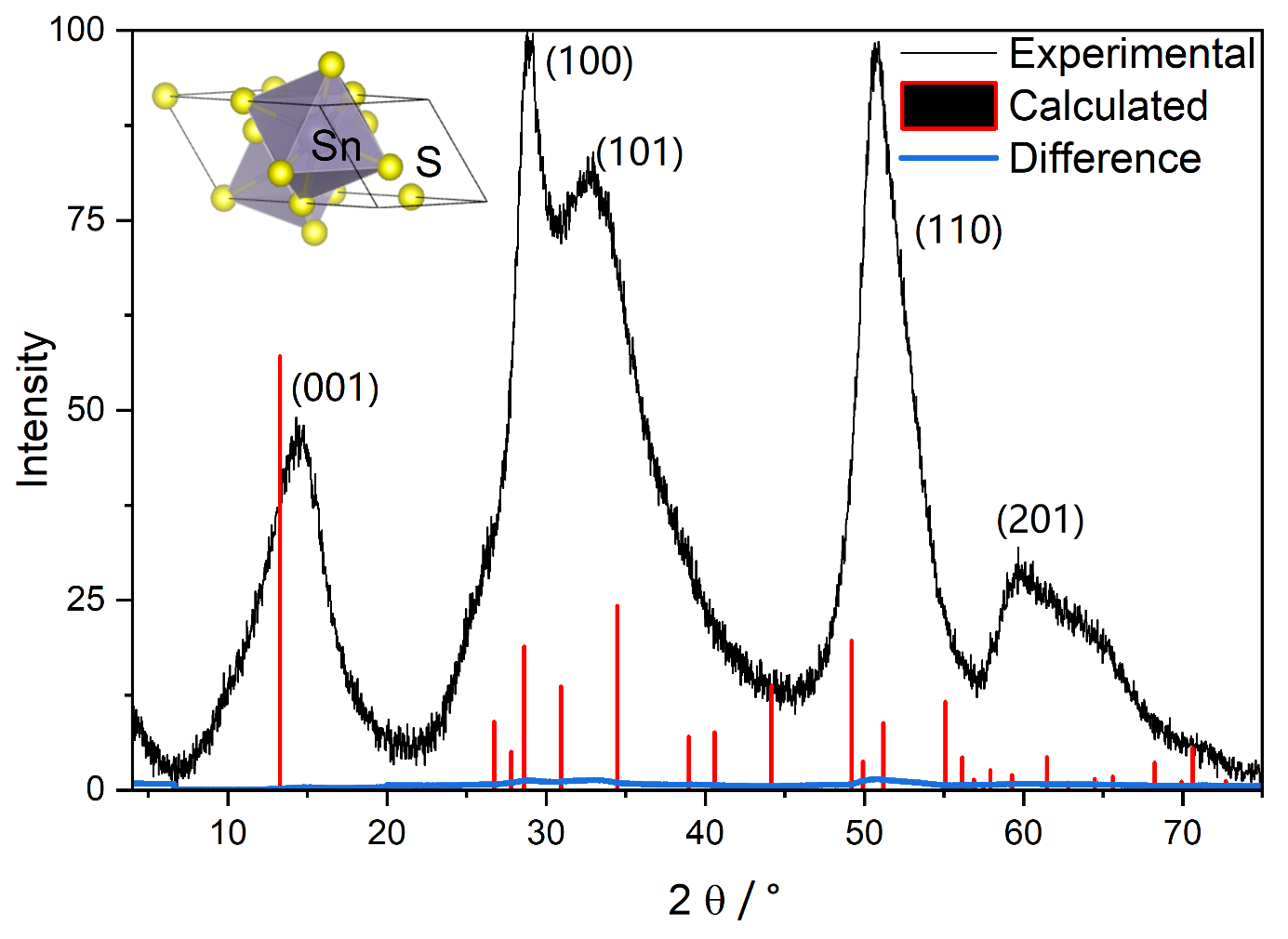


**Supplementary Figure S3.** XRD data of 2D SnS_2_ with the results of a Rietveld analysis showing the difference to the fit calculated according to: Sy=∑_i_(w_i_(y_i_(obs)-y(calc))^2^, where S is the intensity of the corrected spectrum, and w is the statistical weight of the experimental and calculated spectra.


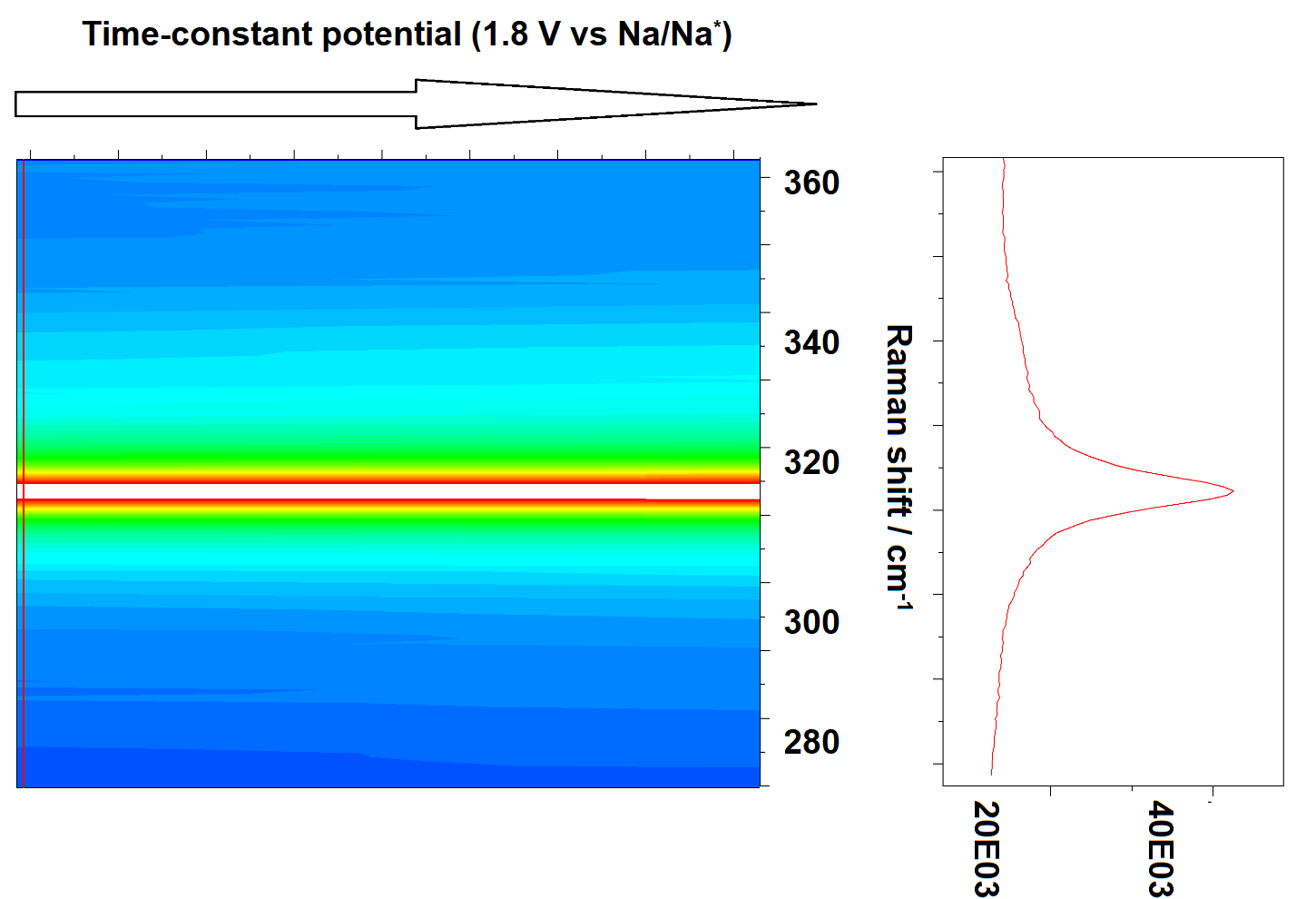


**Supplementary Figure S4.** Coherence of the A_1g_ signal during the sodiation acquired in order to exclude laser heating effects upon cycling with time-constant potential. No changes in the Raman intensity nor the FWHM are observed over two hours period.


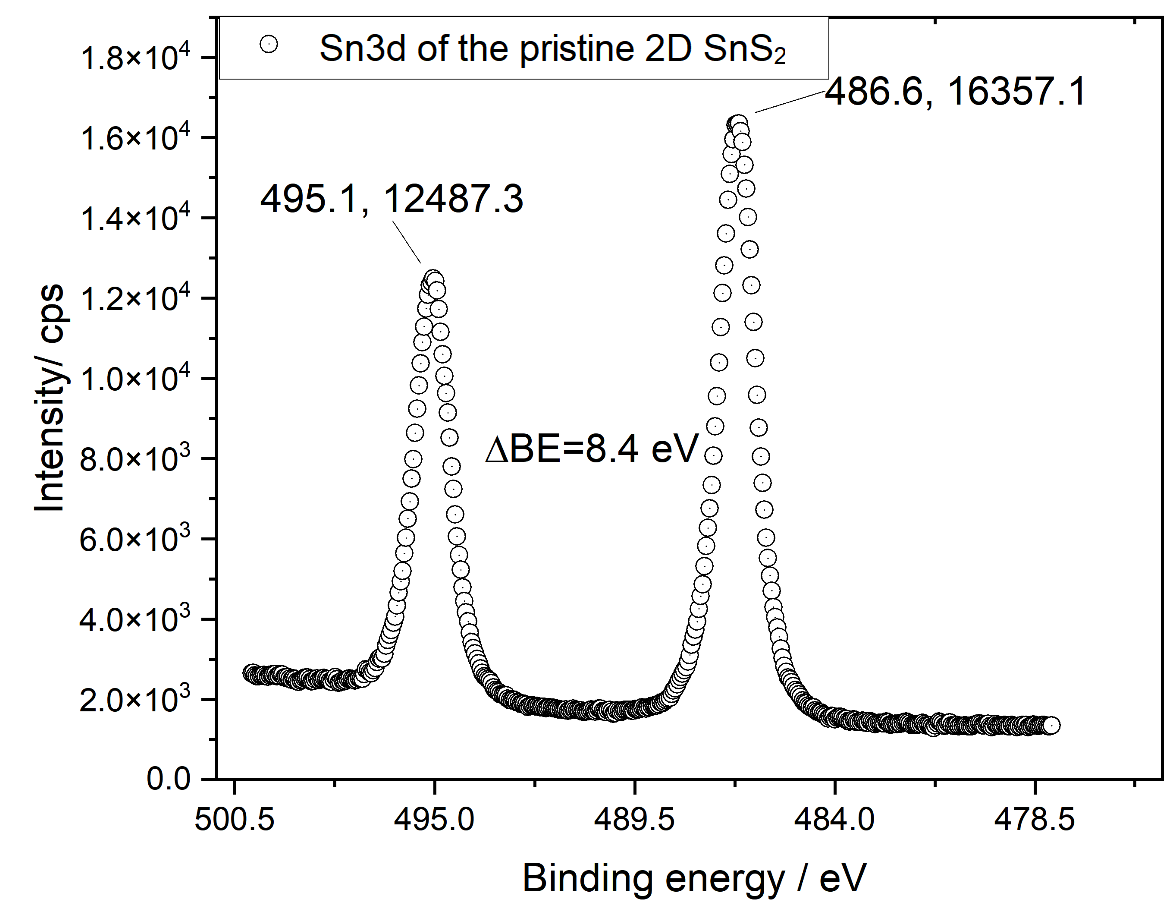


**Supplementary Figure S5.** Sn3d photoemission of the pristine 2D SnS_2_ prior metalation.


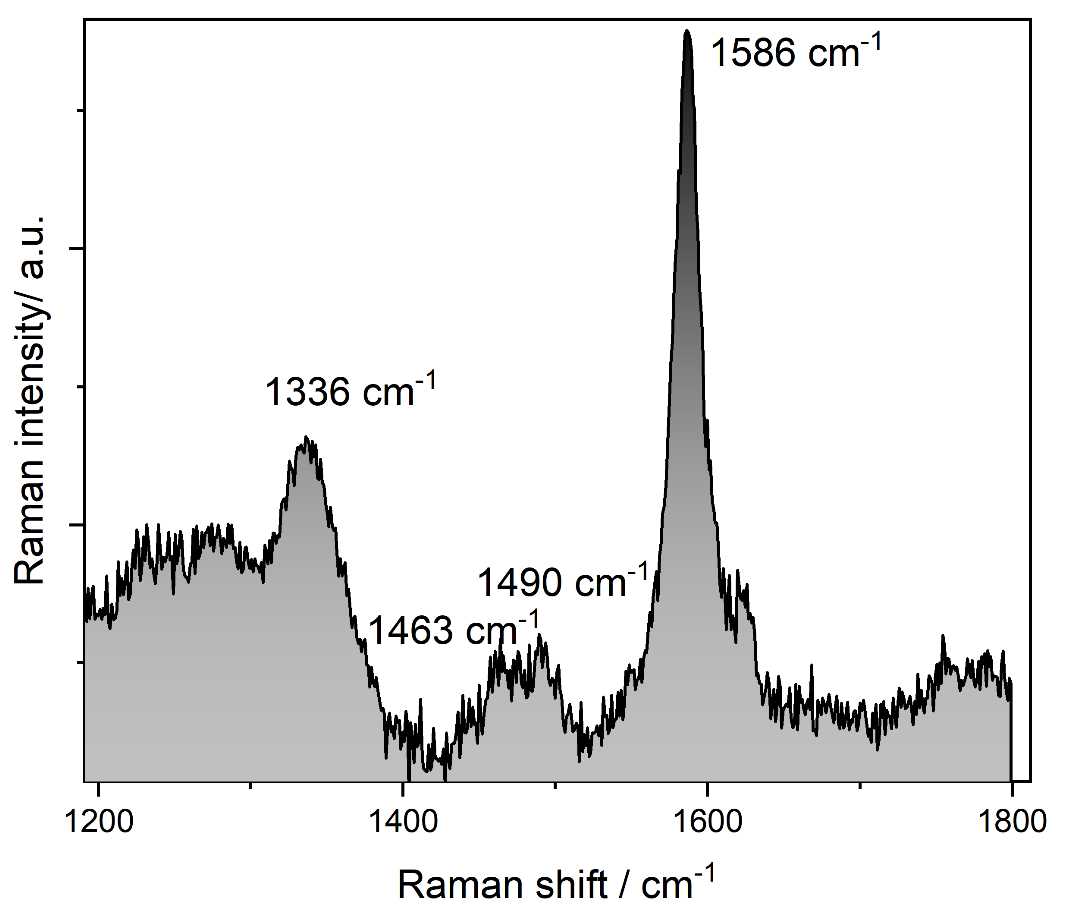


**Supplementary Figure S6.** Cut-off of the experimental *operando* Raman spectra (633 nm) at 0.6V vs Li/Li^+^ of 2D SnS_2_ in a LIB battery, suggesting the presence of the non-solvated anionic CO_2_ radical (O=C^·^-O−) at 1336 cm^-1^ [12]. Based on the literature data, the proposed reaction is as follows: LiSn(I) + 2 O=C^·^-O^−^ + 2Li_2_S → LiSn(III)S_2_ + 2 CO_2_(gas). Please note that O=C^·^-O− is the electron withdrawing source; as a radical, its oxidation potential and reactivity can be expected. Li_x_SnS_2_ is blue-shifted, which confirms the presence of the anionic radical [4]. The bands at 1463 cm^-1^, 1490 cm^-1^ and 1586 cm^-1^ correspond respectively to formate ions from electrolyte decomposition, side-band of the anionic O=C^·^-O− species and CO asymmetric stretching.

**
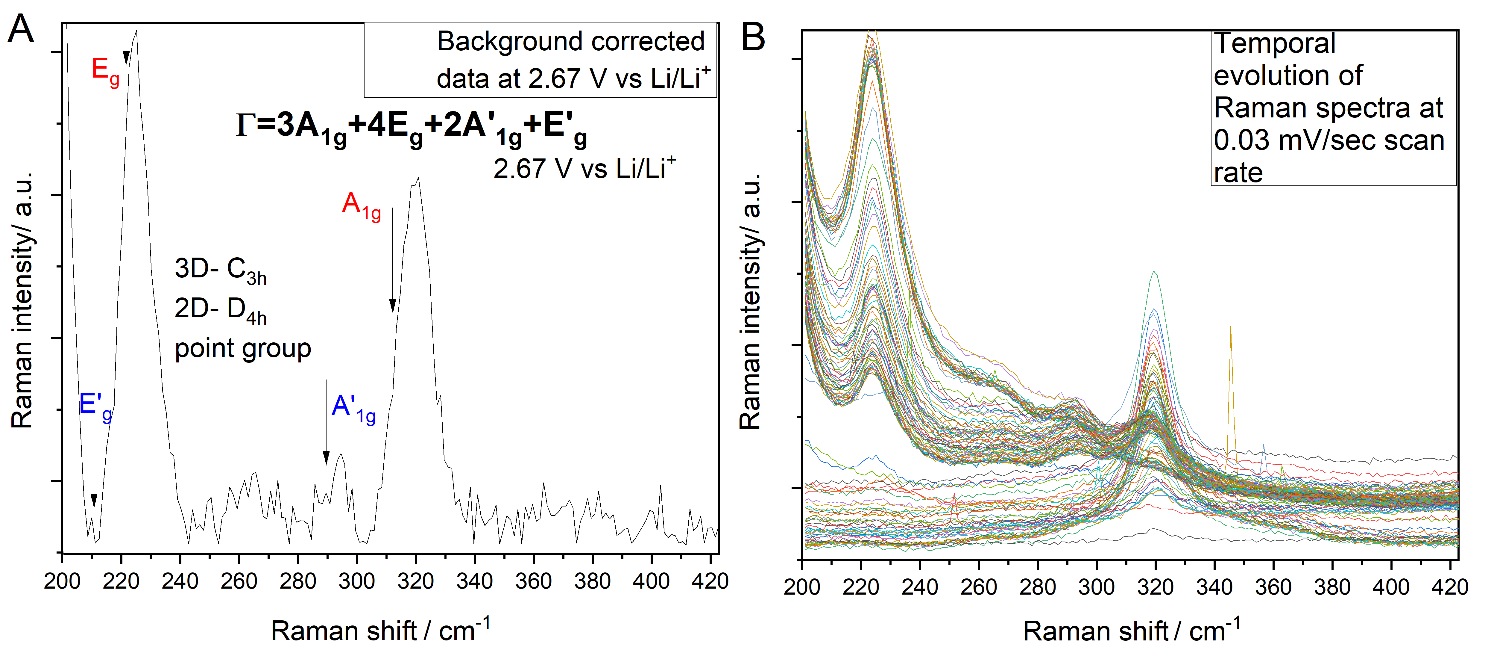
Supplementary Figure S7. (A)** Symmetry-adapted linear combination (SALC) determination of the mixture of octahedral (red: Sn^III^) and tetrahedral (blue: Sn^IV^) contributions to the Raman spectra. The arrows indicate the representative Raman active mode. The ratios of integrals beneath the active modes are consistent with the theoretical assumptions posed by the irreducible representation Γ. **(B)** Stepwise evolution of *operando* Raman spectra towards generation of the Sn(III) oxidation state recorded for 2D SnS_2_ in LIB cell with shorter Raman acquisition time every 30 seconds at a scan-rate of 0.03 mV/sec. The Sn(III) contribution grows and subsequentially again fades away. Please mind the different point groups for different SnS_2_ dimensionality.


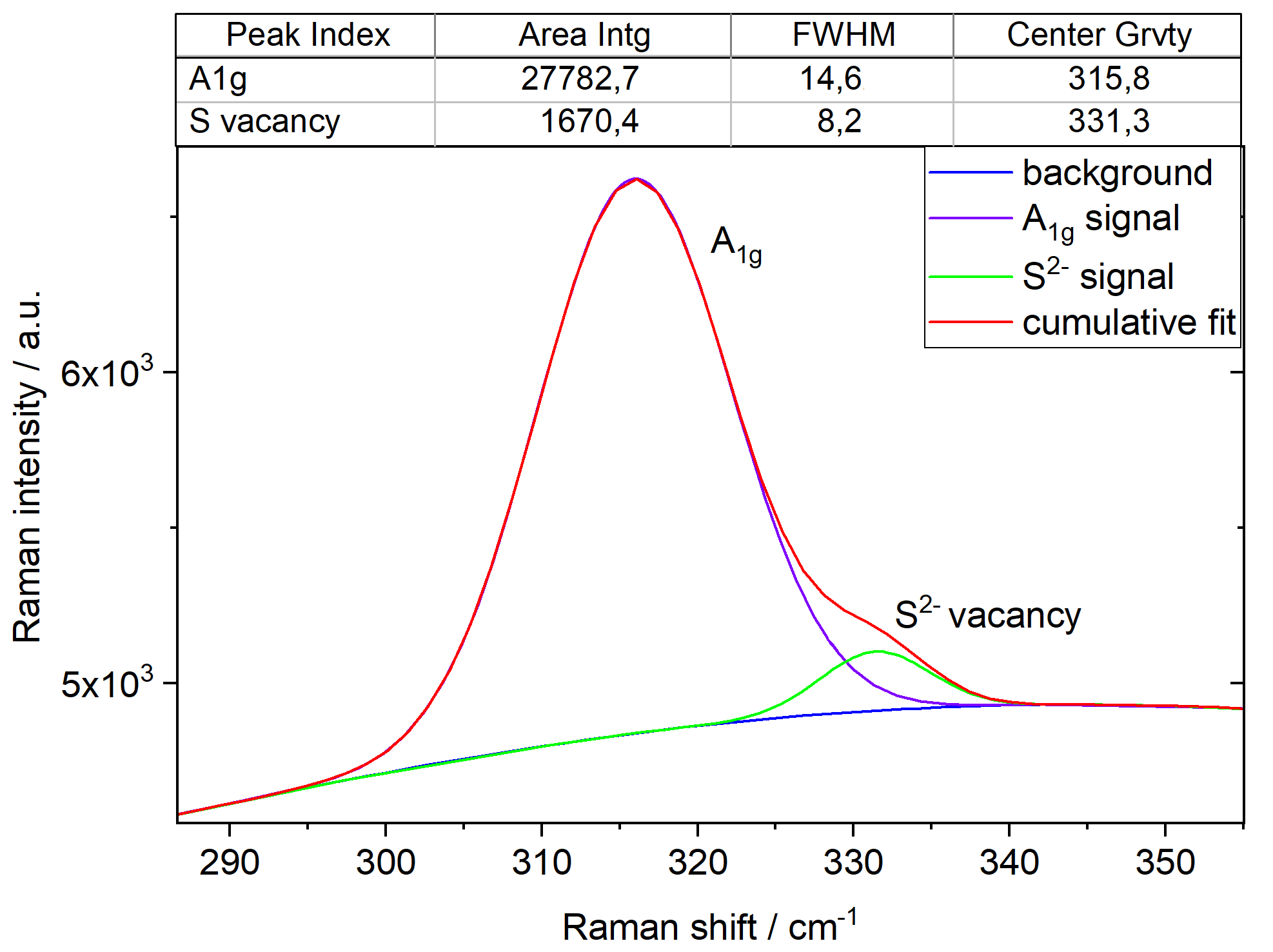


**Supplementary Figure S8.** Raman spectrum (633 nm) of 2D SnS_2_, indicating the formation of S^2-^ vacancies within 2D SnS_2_ at 1.5 V vs Li/Li^+^. As several reports indicated the S^2-^ vacancy to overlap with the Raman inactive A_1u_ mode, due to the use of non-resonant 633 nm red-laser excitation, we exclude the presence of A_1u_ in our study.


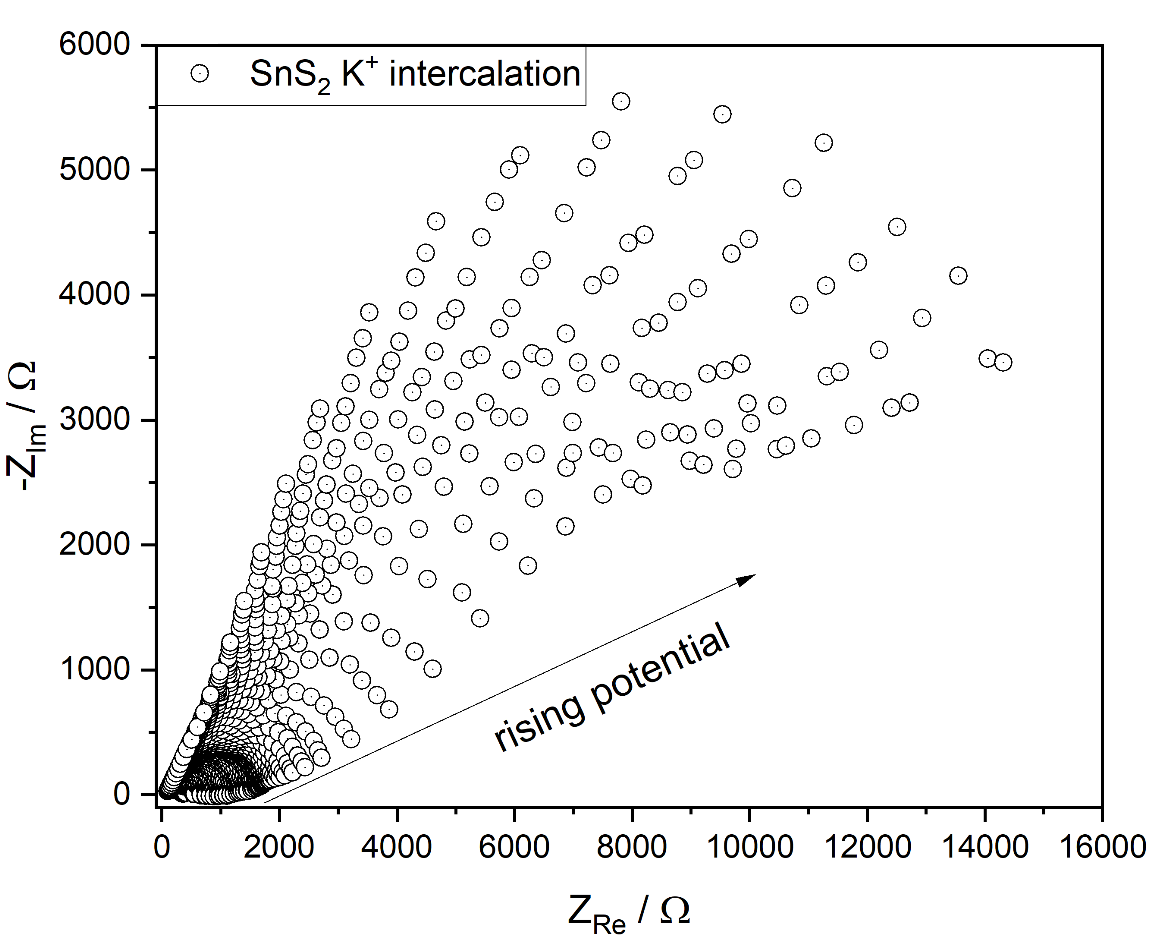


**Supplementary Figure S9.** SPEIS plot with the rising potential for K^+^ intercalation within SnS_2_.


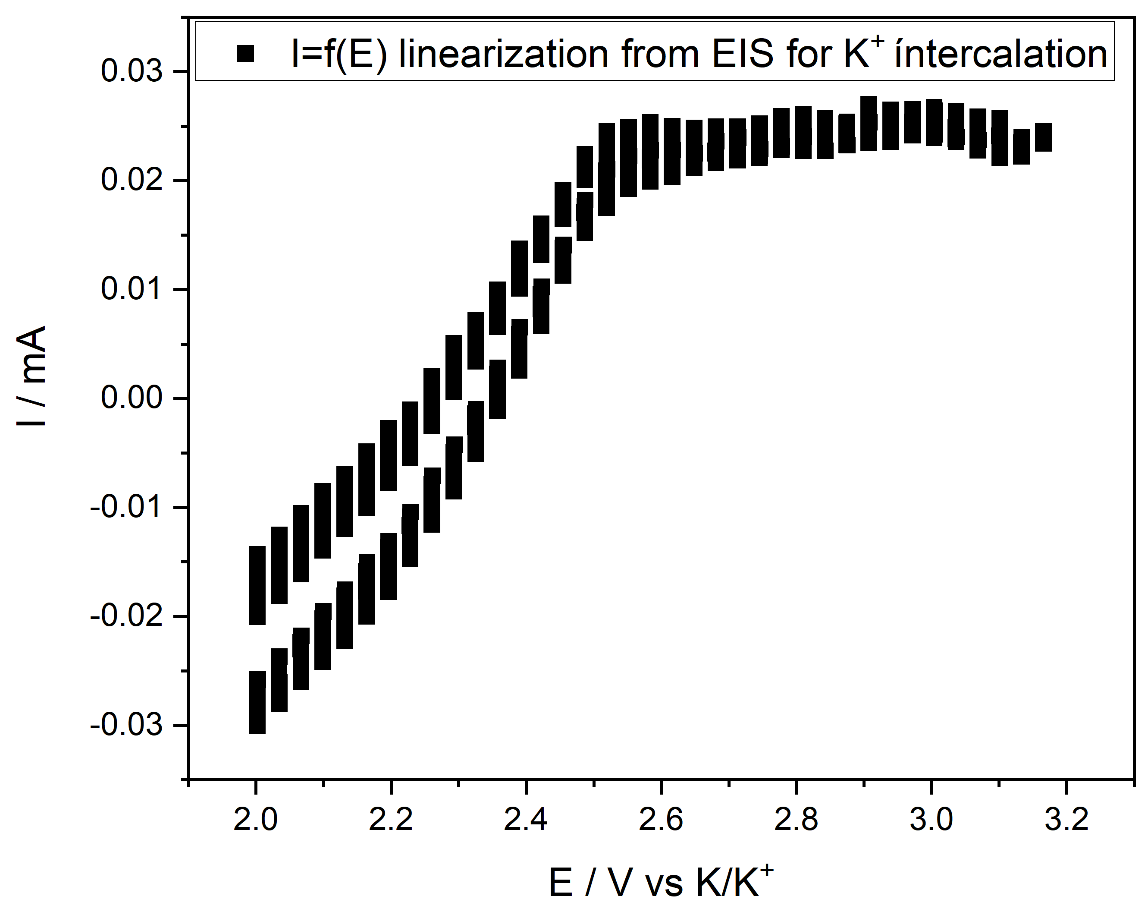


**Supplementary Figure S10.** I = f(E) linearization plot from the EIS experiment, acquired in order to present the CV-behavior of the K^+^ intercalation.


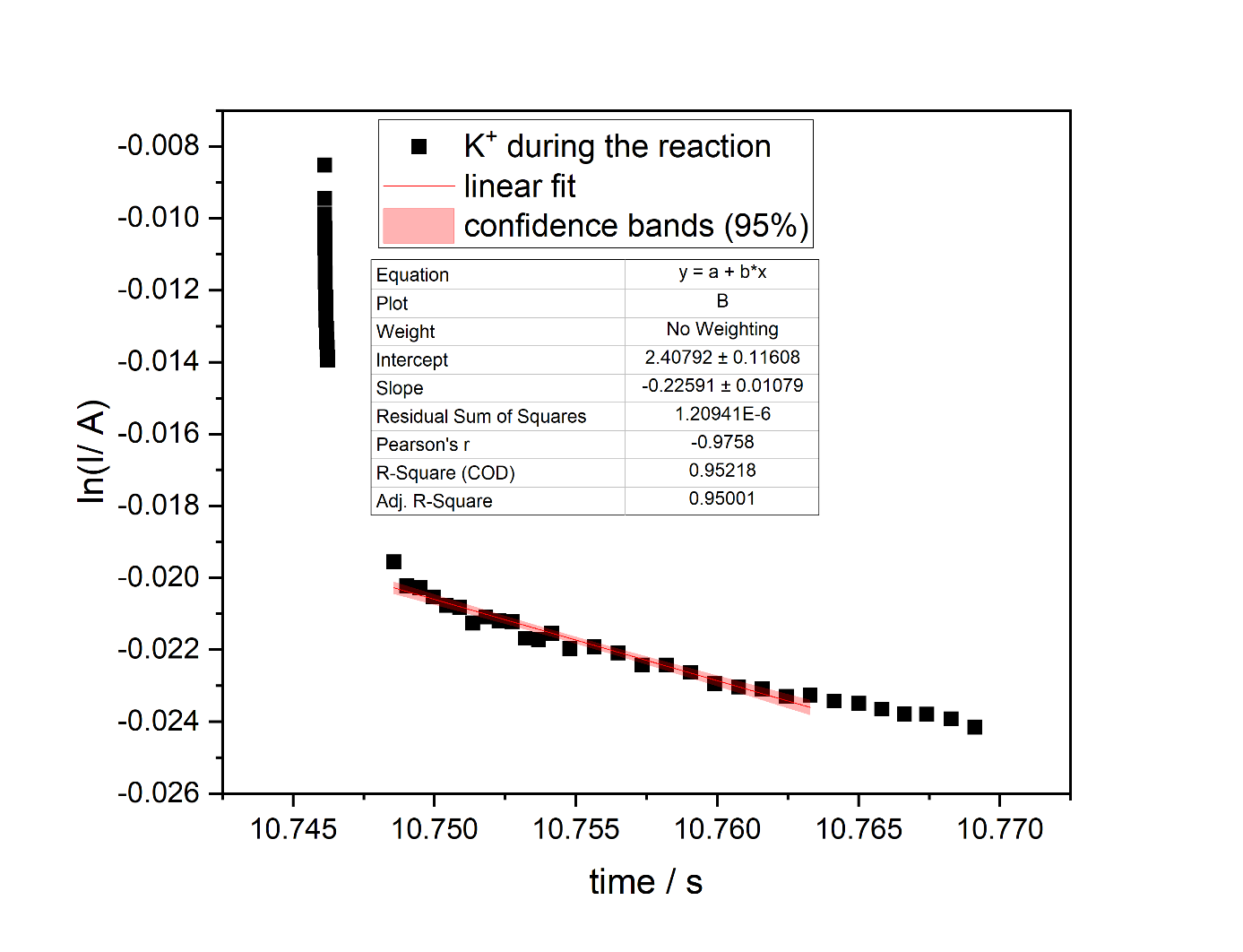


**Supplementary Figure S11.** Current drop in time used for estimation of the diffusion coefficient (D) in Table 1 according to the equation d(ln i)/dt = D π^2^/ 4L^2^ (L = 0.5 cm, t = time, L = pellet thickness).


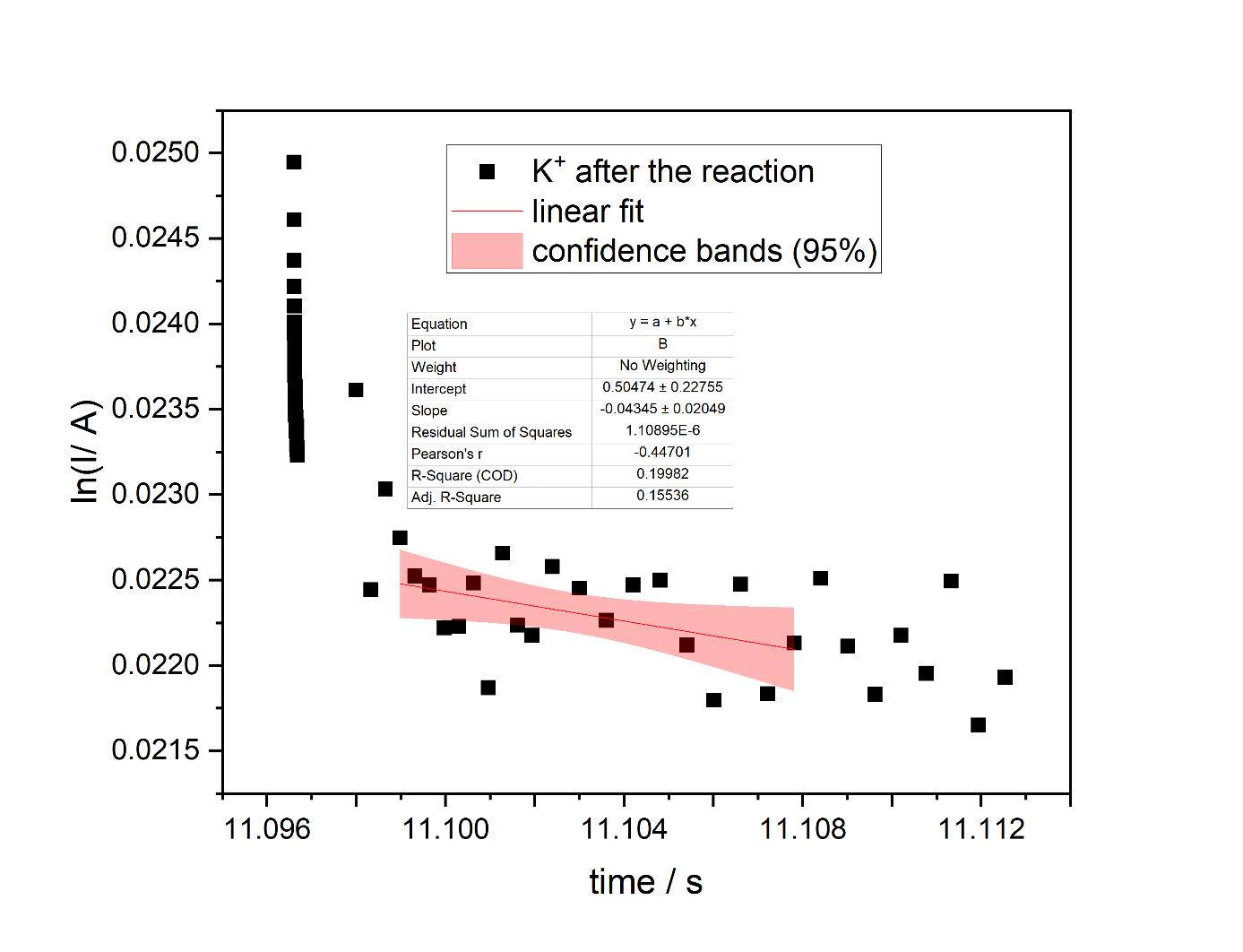


**Supplementary Figure S12.** Current drop in time used for estimation of the diffusion coefficient (D) in Table 1 according to the equation d(ln i)/dt = D π^2^/ 4L^2^ (L = 0.5 cm, t = time, L = pellet thickness).


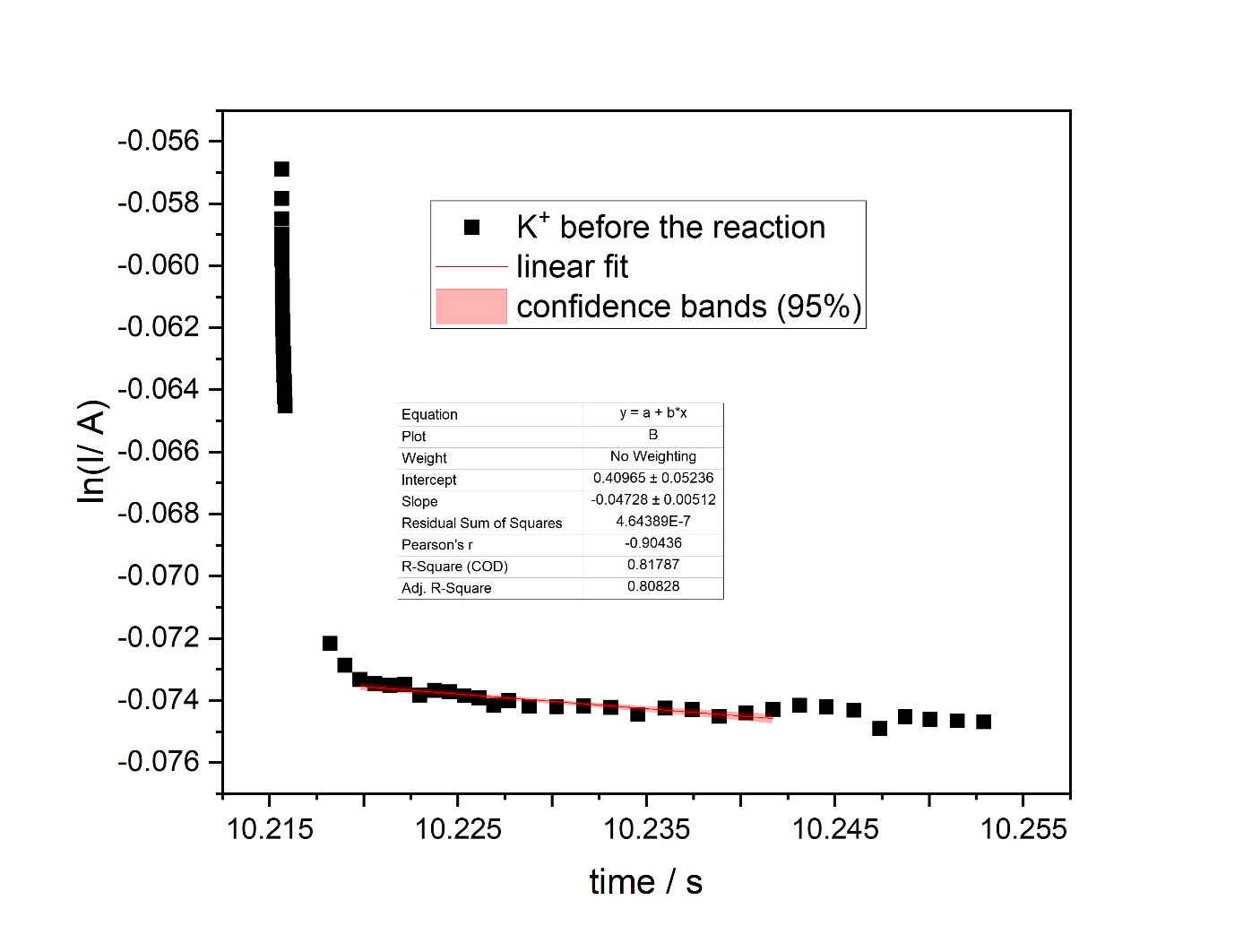


**Supplementary Figure S13.** Current drop in time used for estimation of the diffusion coefficient (D) in Table 1 according to the equation d(ln i)/dt = D π^2^/ 4L^2^ (L = 0.5 cm, t = time, L = pellet thickness).


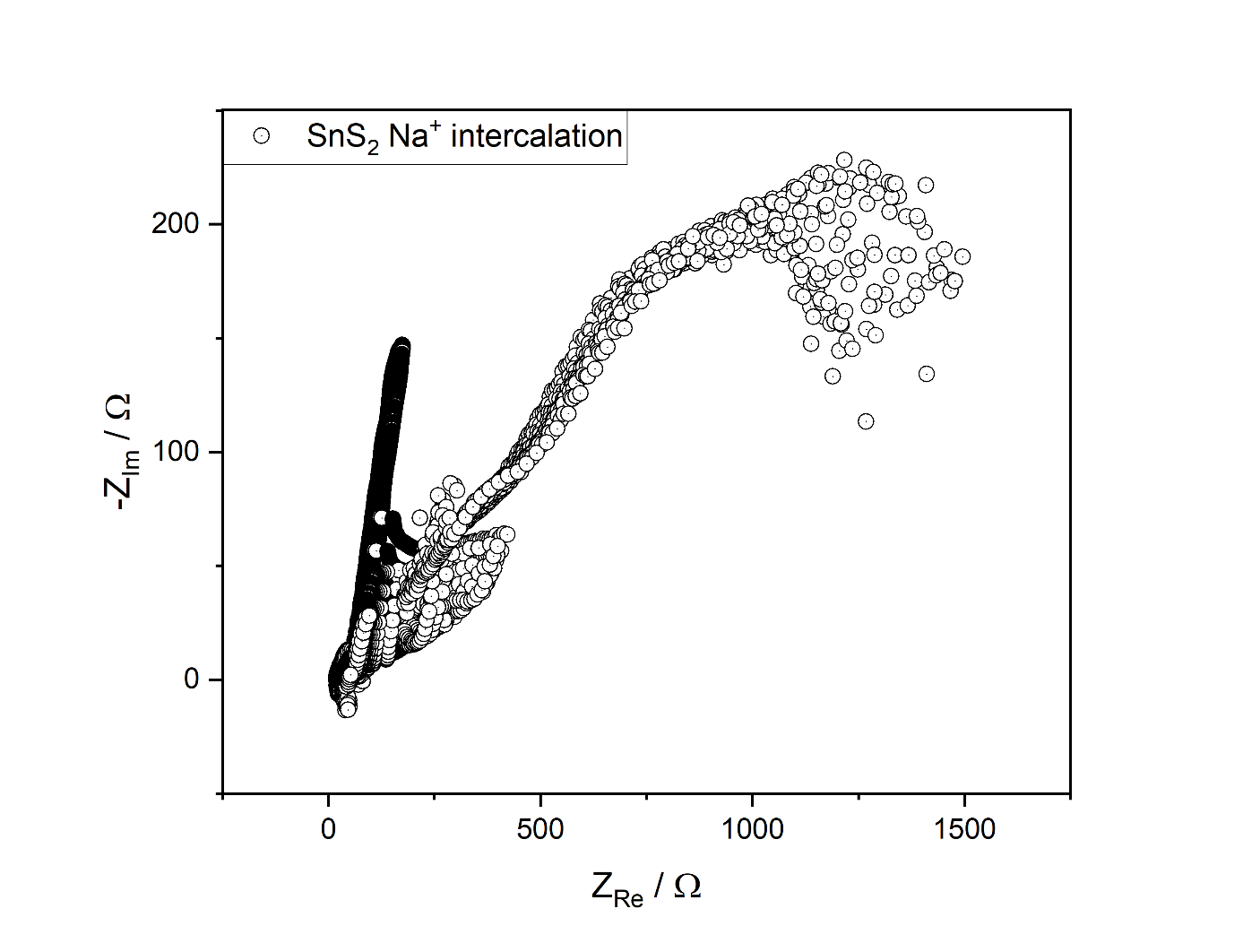


**Supplementary Figure S14.** SPEIS plot with the rising potential for Na^+^ intercalation within SnS_2_.


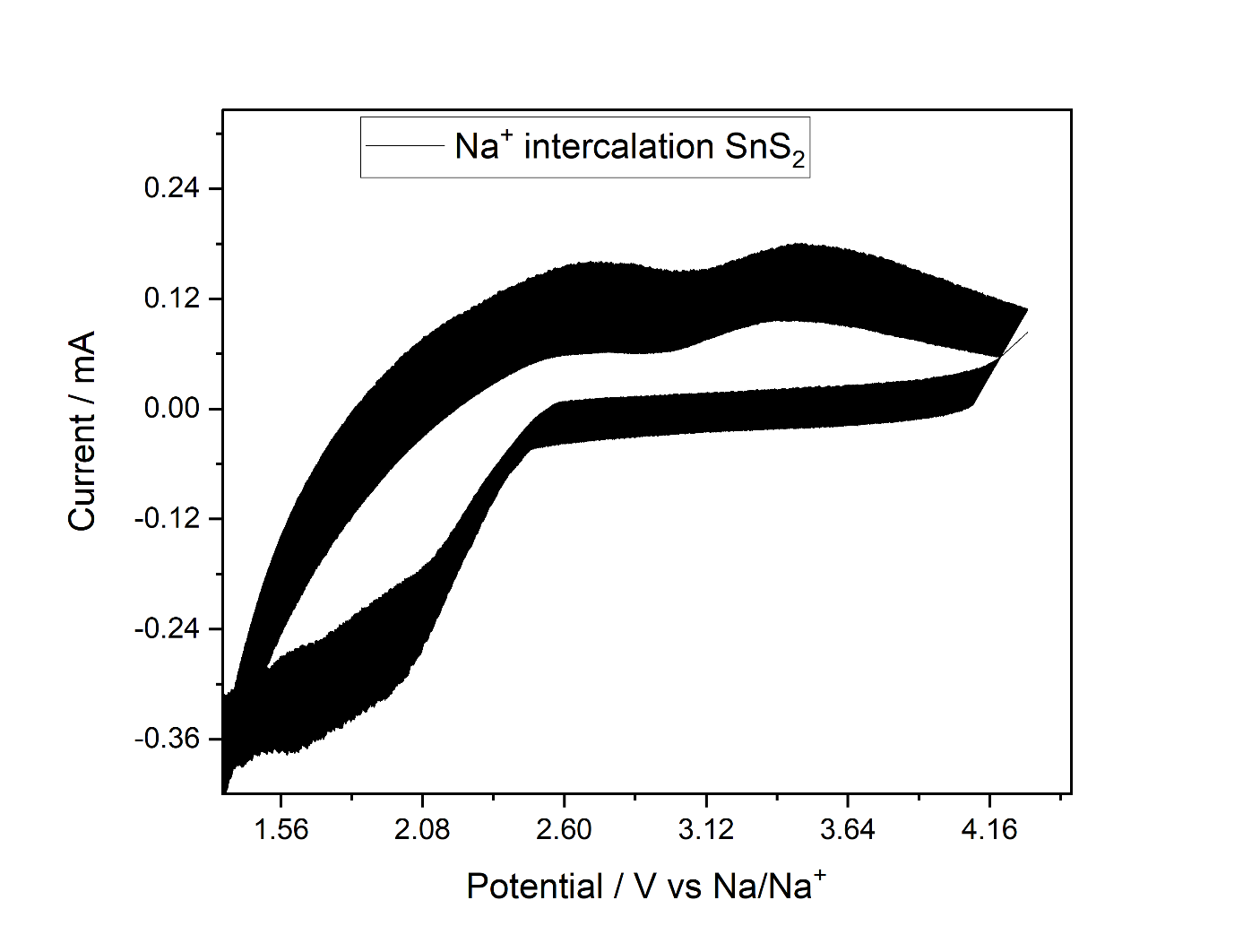


**Supplementary Figure S15.** DPV plot of the Na^+^ intercalation within 2D SnS_2_.


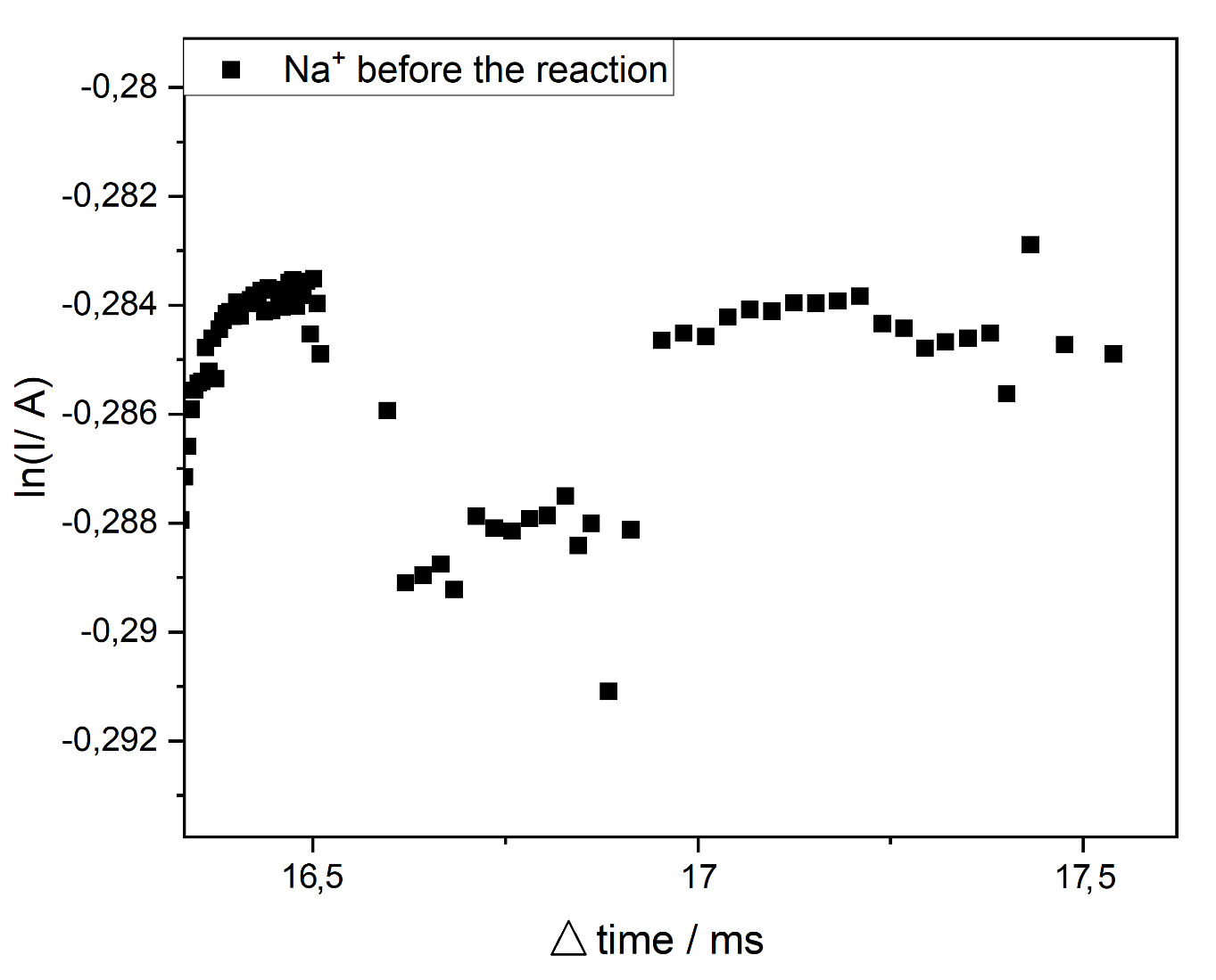


**Supplementary Figure S16**. Close-cut off current drop in time used for estimation of the diffusion coefficient (D) in Table 1 according to the equation d(ln i)/dt = D π^2^/ 4L^2^ (L = 0.5 cm, t = time, L = pellet thickness). Please note the small time-scale of the process. In order to retain clarity, time difference is plotted.


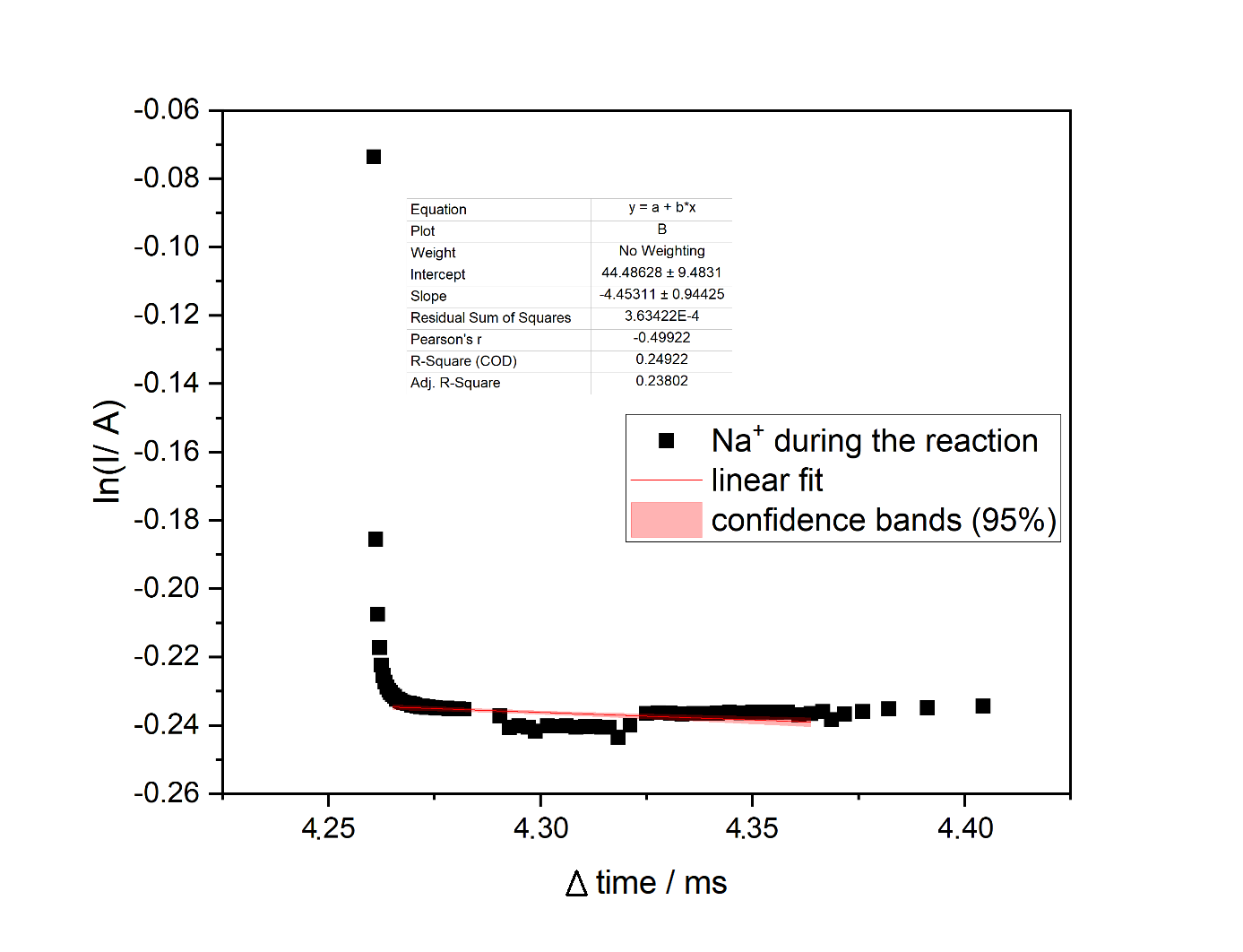


**Supplementary Figure S17.** Current drop in time used for estimation of the diffusion coefficient (D) in Table 1 according to the equation d(ln i)/dt = D π^2^/ 4L^2^ (L = 0.5 cm, t = time, L = pellet thickness). Please note the small time-scale of the process. In order to retain clarity, the time difference is plotted.


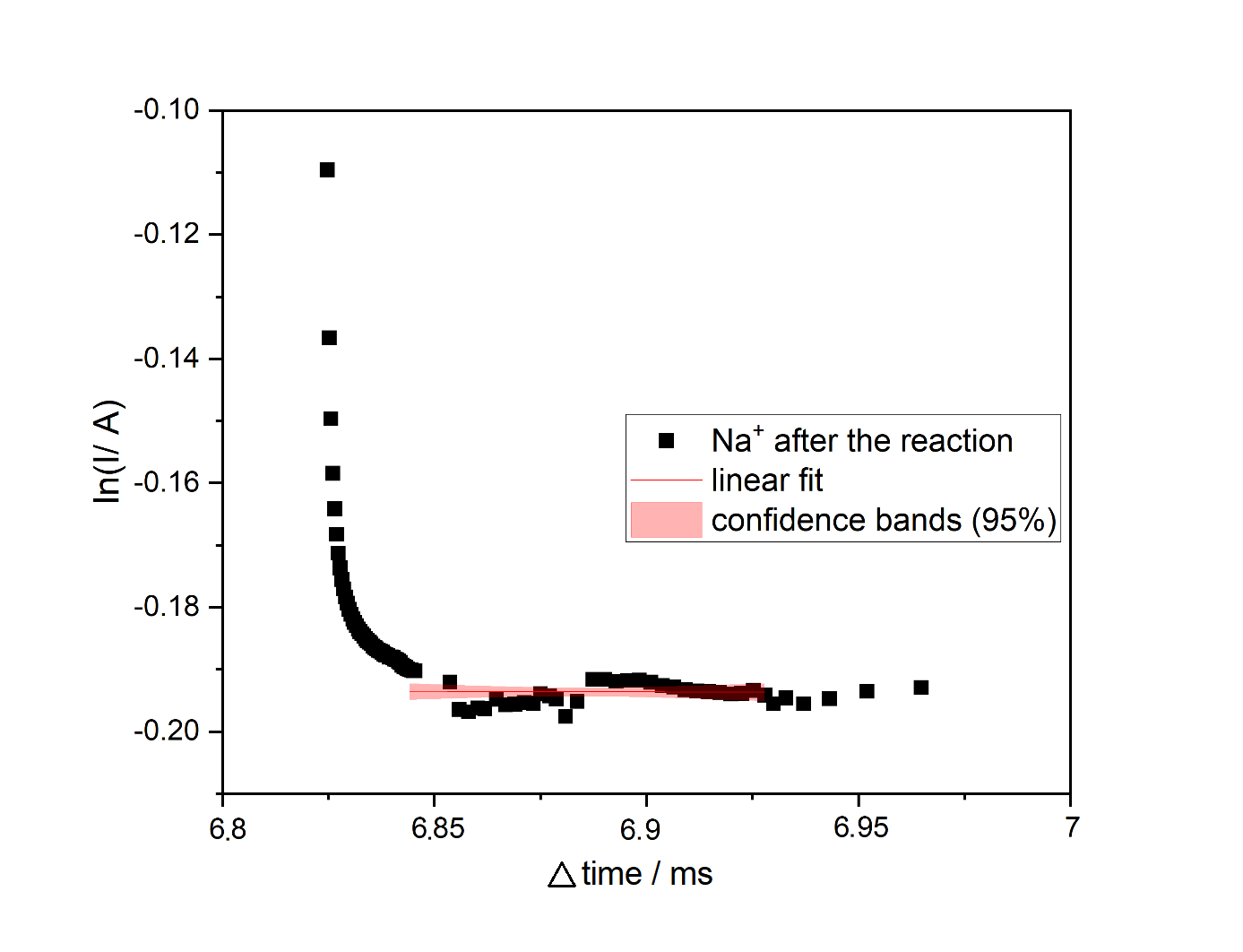


**Supplementary Figure S18.** Current drop in time used for estimation of the diffusion coefficient (D) in Table 1 according to the equation d(ln i)/dt = D π^2^/ 4L^2^ (L = 0.5 cm, t = time, L = pellet thickness). Please note the small time-scale of the process. In order to retain clarity, the time difference is plotted.


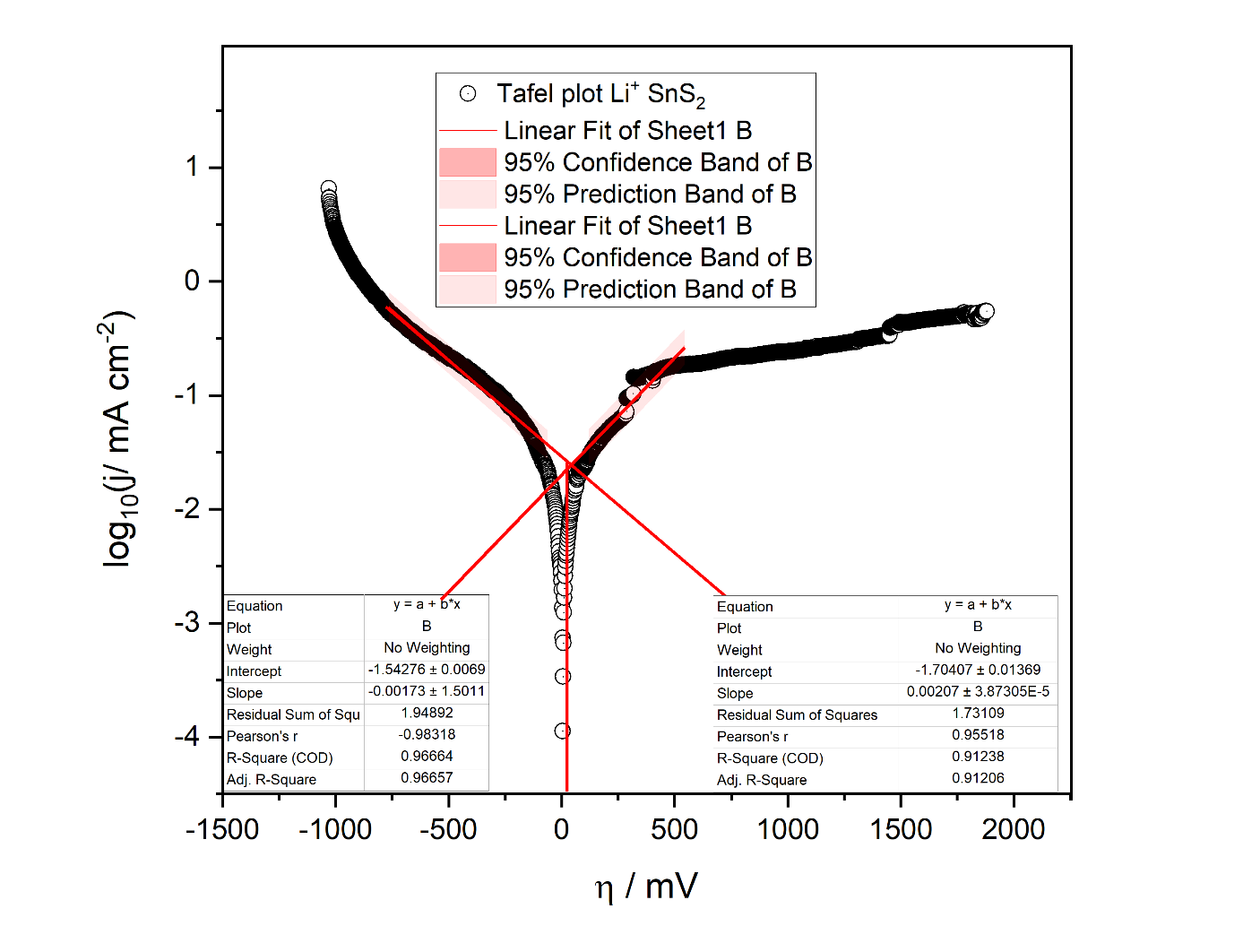


**Supplementary Figure S19.** Exemplary Tafel plot used to extract the charge-transfer merits summarized in Table 3 applying the potential range and conditions specified in materials and methods.


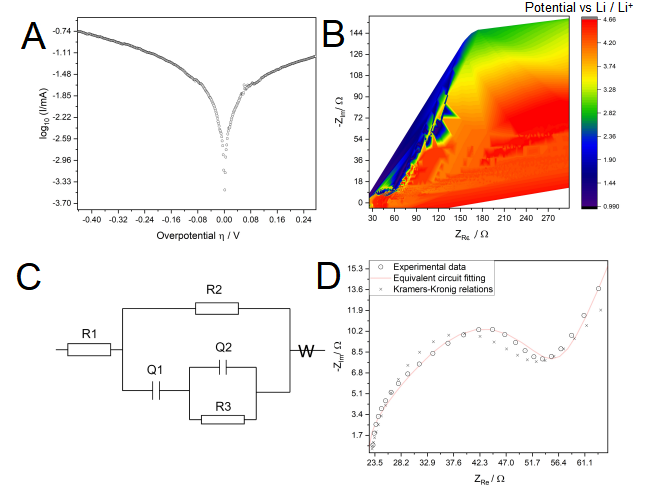


**Supplementary Figure S20.** **A** Tafel-plot of SnS_2_ lithiation and **B** potential-resolved impedance spectroscopy with **C** equivalent circuit used for the analysis shown in Table 1 (R1: solution resistance; R2, R3: material resistance; Q1, Q2: material capacitance; W: Warburg ion diffusion). **D** PEIS scan at the reaction maximum (2.72 V vs Li/Li^+^) with fitting results and validation of the impedance spectrum by Kramers-Kronig relations (3% error).


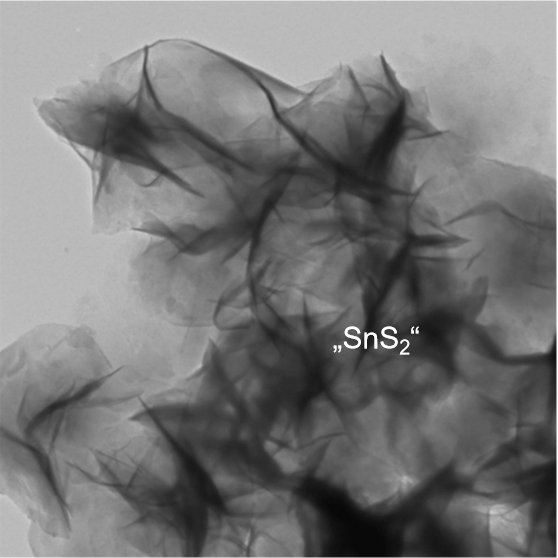


**Supplementary Figure S21.** TEM image of 2D SnS_2_.


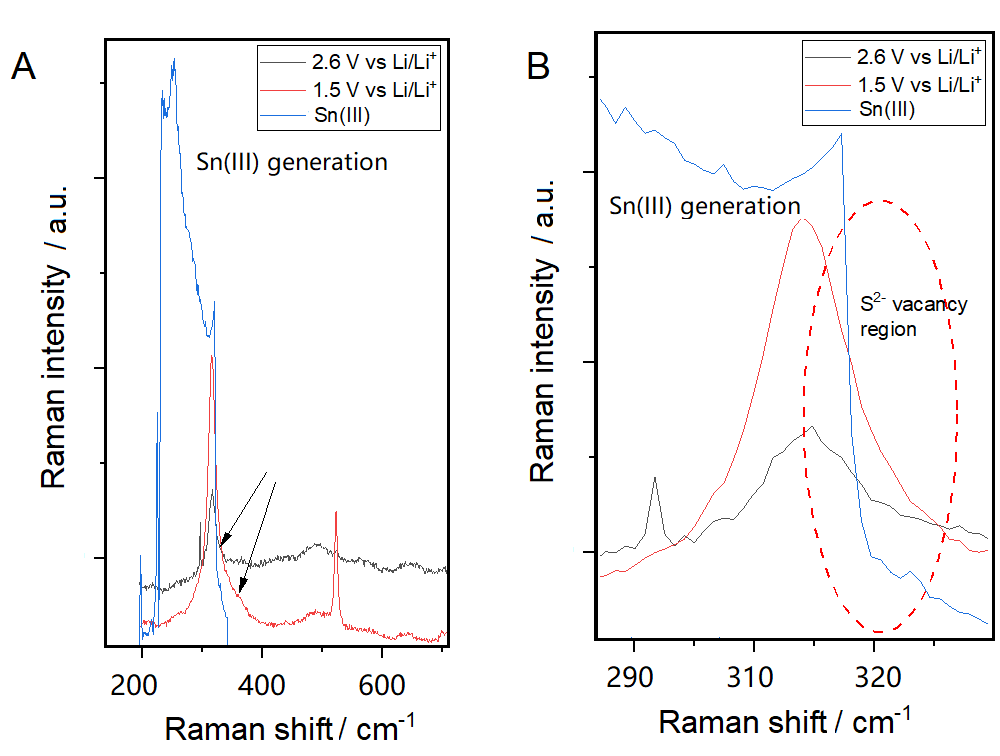


**Supplementary Figure S22.** **(A)** Raman spectra recorded at 2.6 vs Li/Li^+^, 1.5 V vs Li/Li^+^ and OCP, indicating the evolution of S^2-^ vacancies prior and after lithiation. (B) Enlarged view of the spectral region around 320 cm^-1^.


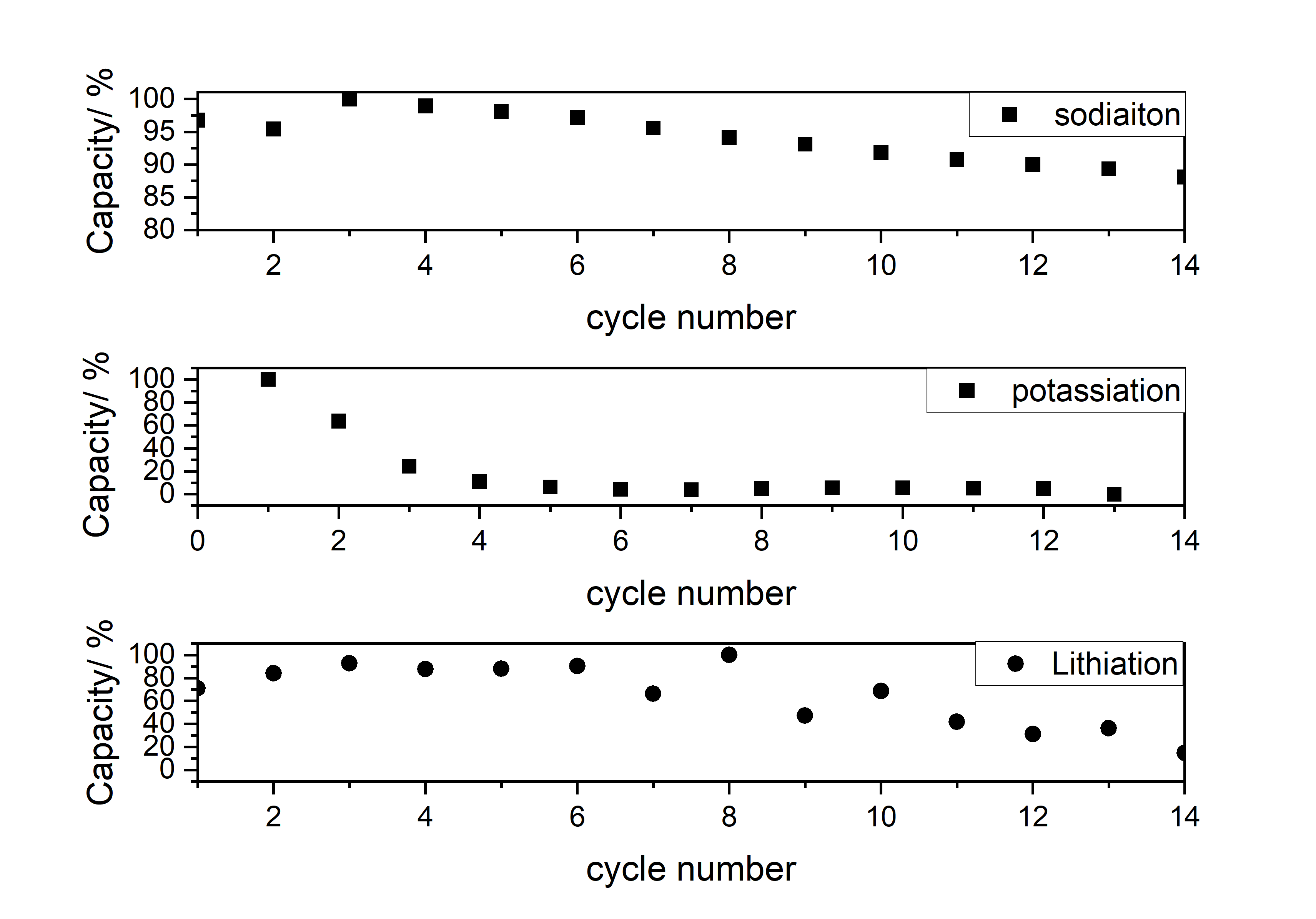


**Supplementary Figure S23.** Scanning performance of LIB, NIB and KIB showing the cycling and rate performance.

**Estimation of the HOMO/LUMO separation based on DPV analysis**

In the following procedure, adapted from [6], no bandgap tuning during the measurement and a steady state was considered. Equation 1 describes the overall procedure for HOMO/LUMO separation:

$\Delta E_{HOMO/LUMO}={ze^{-}\cdot[(E}_{Ox vs SHE}-E_{Red vs SHE})- 3.022 V]+4.4 eV$ (1)

where z means the number of the electrons being transferred during the RedOx reaction, E_ox_/E_Red_ are respective potentials of the observed reactions versus standard hydrogen electrode (SHE) and 4.4 is the difference between the vacuum level and the potential of SHE: ϕ_NHE_ = - 4,85 - ϕ_Vac_.

For further evaluation of the material conductivity and applicability as an anode, we performed series of electrochemical tests. A PITT experiment was used to evaluate the ionic diffusion coefficient, a Tafel analysis was used to evaluate the kinetics of the lithiation process, while SPEIS was performed to evaluate the electron mobility within the cell. Figure S17 A shows the Tafel analysis with a characteristic dip at 0 V overpotential. The anodic part of the charge-transfer proportionality constant α of the lithiation reaction evaluated by the Tafel experiment was established as 0.68 and the cathodic part was 0.32. This corresponds well to the fact, that anodic oxidation and electron stripping are the major contributions to the lithiation. Figure S17 B depicts potential-resolved electrochemical impedance scans, showing the distinctive transition of the capacitive part (see Figure S17 C) to the diffusion regime at 3.74 V. This behavior originates from the saturation with lithium and a pure ion-diffusion process (red spheres) of that part (prevalent Warburg impedance).

**Table S1** XPS analysis of 2D SnS_2_ prior and after intercalation.

| Signal | SnS_2_ [at%] | SnS_2_/Li^a^  [at%] | SnS_2_/Na^a^ [at%] |
| --- | --- | --- | --- |
| Sn3d | 9.6 | 2.7 | 1.4 |
| S2p | 10.9 | 2.4 | 1.5 |
| Li1s | - | 12.3 | - |
| Na1s | - | - | 1.0 |
| K2p | - | - | - |
| C1s | 70.5 | - | 54.9 |
| O1s | 7.92 |  | 70 |
| Sn:S | 1.08 | 1.25 | 1.14 |
| Sn:Li:S | - | 1.0:1.1:4.5 | - |
| Sn:Na:S | - | - | 1.4:1.0:1.5 |
| Sn:K:S | - | - | - |

^a^ performed post-mortem in a charged state of 3.6 V vs M/M^+^ (M = Li/Na)

**Proposed Analysis of the defects generated during the lithiation by Kröger-Vink notation**

Figure S20 shows the evolution of features, which may be attributed with sulfur vacancies, obtained after A_1g_ Raman active band deconvolution at 1.5 V vs Li/Li^+^. The generation of sulfur vacancies may be associated with two findings. Here we propose a possibility of how those vacancies came to be. Firstly, the reaction of SnS_2_ with Li_2_S and metallic Sn at 1.5 V vs Li/Li^+^ in the anodic scan. Secondly, the Schottky and Frenkel defect generation within alloyed tin from SnS_2_ according to the following Kröger-Vink notation:

Sn_Sn_^x^ → V_Sn_^‘‘‘‘^ + Sn_i_ ^....^  (1)

S_S_^x^ → V_S_^..^ + S_i_^´´^ (2)

Sn_Sn_^x^ + 2S_S_^x^ → V_Sn_^‘‘‘‘^ + 2V_S_^..^ + SnS_2_ (3)

where S stands for sulfur, Sn for tin, V for vacancy, ´ is the positive charge, ^.^ is the negative charge, i means „isolated“ state, and x describes no change in the oxidation state [7]. In the initial state at room temperature the defects V_Sn_‘‘‘‘ and V_S_^..^ may possibly be associated with the features at 328 cm^-1^ and 344 cm^-1^ in Figure 5 [8].

Defect features visible initially in Raman spectra may be due to sulfur vacancies from the imperfect synthesis, while during the metalation with lithium/sodium and potassium additional defects are being created. The V_S_^2^ and V_Li_^-^ within 2D SnS_2_ may be associated with the features at 319.5 cm^-1^ and 95.1 cm^-1^. The metalation induced defects are formed according to:

xLi_Li_^x^ + Sn_Sn_^x^ + 2S_S_^x^ → xV_Li_ ^.^ + V_Sn‘‘‘‘_ + 2V_S_^..^ + Li_x_SnS_2_ (4)

The full lithiation and observed changes, alongside with phase transformations induced by electric field and metalation, are described in the section dealing with the spectroelectrochemical analysis. The creation of Sn_2_S_3_ was not observed spectroscopically in our studies.

**Supplementary Table S2.** Kröger-Vink notation of the changes occurring within the anodic and cathodic scans [9] [10].

| Defect | Raman position^1^/ Potential^2^ |
| --- | --- |
| Sn_Sn_^x^ | 95.1^e^ / 1.42 V |
| S_S_^x^ | 59.1^d, e^ / 1.42 V |
| V_Sn_^‘‘‘‘^ | 716.5 / 1.09:2.69 V^b^ |
| Sn_i_ ^...^ | 95.1^e^ / 1.42 V |
| V_S_^..^ | 319.5^a^ / OCP |
| S_i_^´´^ | 319.5: 59.1^d, e^ / 1.57 - 2.94 V |

^1^ During *operando* scan with 633 nm laser excitation.

^2^ vs Li/Li^+^ OCP = open circuit potential.

^3^ In selected cases closest speculative selection.

^a^ Deconvoluted signal

^b^ No signals for the tin-vacancy were observed in between those potentials

^c^ We postulate that those vacancies might be observed at cryogenic temperatures [11].

^d^ Signal indistinguishable from the also possible Sn_2_S_3_.

^e^ Band-edge emission of the SnS_2_ and Sn_2_S_3_.

**References:**

| [1] M. Radtke, A. Ignaszak, Classical group theory adapted to the mechanism of Pt_3_Ni nanoparticle growth: the role of W(CO)_6_ as the “shape-controlling” agent, Phys. Chem. Chem. Phys., 2015, 18, 75, https://doi.org/10.1039/C5CP05060J . |
| --- |
| [2] M. Goehring, W. Koch, Notizen: Derivate des Heptaschwefelimids, S_7_NH, Z. Naturforsch., 1952, 7b, 634, https://doi.org/10.1515/znb-1952-1111.  [3] C. S. Lu, J. Donohue, An electron diffraction investigation of sulfur nitride, arsenic disulfide (realgar), arsenic trisulfide (orpiment) and sulfur, J. Amer. Chem. Soc., 1944, 66, 818, https://doi.org/10.1021/ja01233a049. |
| [4] K. Ikeda, S. Terada, T. Manadi, K. Ueno, K. Dokko, M. Watanabe, Lithium-tin alloy/sulfur battery with a solvate ionic liquid electrolyte, Electrochem., 2015, 83, 914, https://doi.org/10.5796/electrochemistry.83.914. |
| [5] T. Sriv, K. Kim, H. Cheong, Low-frequency Raman spectroscopy of few-layer 2H-SnS_2_, Sci. Rep., 2018, 8, 10194, https://doi.org/10.1038/s41598-018-28569-6. |
| [6] R. Hausbrand, Electronic energy levels at Li-ion cathode–liquid electrolyte interfaces: Concepts, experimental insights, and perspectives, J. Chem. Phys., 2020, 04, 180902, https://doi.org/10.1063/1.5143106. |
| [7] K. Navaratnarajah, A. L. Solovjov, R. Vovk, A. Chroneos, Defects, diffusion and dopants in Li_8_SnO_6_, Heliyon, 2021, 7, e07460, https://doi.org/10.1016/j.heliyon.2021.e07460. |
| [8] J. M. Skelton, L. A. Burton, A. J. Jackson, F. Oba, S. C. Parker, A. Walsh, Lattice dynamics of the tin sulphides SnS_2_, SnS and Sn_2_S_3_: vibrational spectra and thermal transport, Phys. Chem. Chem. Phys., 2017, 27, 12452, https://doi.org/10.1039/C7CP01680H. |
| [9] J. D. Prades, J. Arbiol, J. R. Morante, M. Avella, L. Zanotti, E. Comini, G. Faglia, G. Sberveglieri, Defect study of SnO_2_ nanostructures by cathodoluminescence analysis: Application to nanowires, Sens. Actuators B, 2007, 126, 6, https://doi.org/10.1016/j.snb.2006.10.014. |
| [10] J. Kaur, J. Shah, R. K. Kotnala, K. C. Verma, Raman spectra, photoluminescence and ferromagnetism of pure, Co and Fe doped SnO_2_ nanoparticles, Ceram. Int., 2012, 7(38), 5563, https://doi.org/10.1016/j.ceramint.2012.03.075.  [11] B. H. Baby, D. B. Mohan, Phase formation study of SnS nanoparticles synthesized through PVP assisted polyol method, IOP Conf. Ser.: Mater. Sci. Eng., 2018, 360, 012003, https://doi.org/10.1088/1757-899X/360/1/012003.  [12] I. Janik, G. N. R. Tripathi, The nature of the CO_2_^-^ radical in water, J. Chem. Phys. 2016, 144154307, https://doi.org/10.1063/1.4946868 |
